# Proteomic Analysis of Hypoxia-Induced Senescence of Human Bone Marrow Mesenchymal Stem Cells

**DOI:** 10.1155/2021/5555590

**Published:** 2021-08-27

**Authors:** Liping Mai, Guodong He, Jing Chen, Jiening Zhu, Shaoxian Chen, Xinghua Hou, Hui Yang, Mengzhen Zhang, Yueheng Wu, Qiuxiong Lin, Min Yang, Xiaohong Li

**Affiliations:** ^1^Research Department of Medical Sciences, Guangdong Provincial People's Hospital, Guangdong Academy of Medical Sciences, Guangzhou 510080, China; ^2^Guangdong Provincial Key Laboratory of South China Structural Heart Disease, Guangdong Cardiovascular Institute, Guangdong Provincial People's Hospital, Guangdong Academy of Medical Sciences, Guangzhou 510080, China

## Abstract

**Methods:**

Hypoxia in hBMSCs was induced for 0, 4, and 12 hours, and cellular senescence was evaluated by senescence-associated *β*-galactosidase (SA-*β*-gal) staining. Tandem mass tag (TMT) labeling was combined with liquid chromatography-tandem mass spectrometry (LC-MS/MS) for differential proteomic analysis of hypoxia in hBMSCs. Parallel reaction monitoring (PRM) analysis was used to validate the candidate proteins. Verifications of signaling pathways were evaluated by western blotting. Cell apoptosis was evaluated using Annexin V/7-AAD staining by flow cytometry. The production of reactive oxygen species (ROS) was detected by the fluorescent probe 2,7-dichlorodihydrofluorescein diacetate (DCFH-DA).

**Results:**

Cell senescence detected by SA-*β*-gal activity was higher in the 12-hour hypoxia-induced group. TMT analysis of 12-hour hypoxia-induced cells identified over 6000 proteins, including 686 differentially expressed proteins. Based on biological pathway analysis, we found that the senescence-associated proteins were predominantly enriched in the cancer pathways, PI3K-Akt pathway, and cellular senescence signaling pathways. CDK1, CDK2, and CCND1 were important nodes in PPI analyses. Moreover, the CCND1, UQCRH, and COX7C expressions were verified by PRM. Hypoxia induction for 12 hours in hBMSCs reduced CCND1 expression but promoted ROS production and cell apoptosis. Such effects were markedly reduced by the PI3K agonist, 740 Y-P, and attenuated by LY294002.

**Conclusions:**

Hypoxia of hBMSCs inhibited CCND1 expression but promoted ROS production and cell apoptosis through activating the PI3K-dependent signaling pathway. These findings provided a detailed characterization of the proteomic profiles related to hypoxia-induced senescence of hBMSCs and facilitated our understanding of the molecular mechanisms leading to stem cell senescence.

## 1. Introduction

Mesenchymal stem cells (MSCs) have become an important resource for cell therapy and regenerative medicine [1] due to their self-renewal capacity, multidirectional differentiation, and low immunogenicity. MSCs are easy to obtain and isolate [2], and they have become attractive donor cells in the field of cell therapy, such as the treatment of hematopoietic and neurodegenerative diseases [3]. Although MSCs proliferate extremely well *in vitro*, after a limited number of passages, they show signs of senescence [4]. Cell senescence is considered a hallmark of aging, accompanied by permanent cell cycle arrest, phenotypic changes, metabolic reprogramming, and secretion of senescence-associated secretory phenotype (SASP) components [5]. The bone mass and osteogenic differentiation capacity of MSCs are significantly reduced when aging occurs. The p53 and Surf1 signaling pathways have been validated as mediating skeletal deformities in the senescence model via affecting the function of MSCs [6]. Promoting MSC proliferation and chondrogenic differentiation in cartilage can repair osteoarthritic lesions via activating the PI3K-Akt signaling pathway [7]. Thus, the senescence of MSCs reduces their proliferation and differentiation abilities, which hinders their therapeutic application [8]. Therefore, it is important to explore the molecular mechanisms of senescence to delay senescence and promote the clinical application of stem cells. Previous studies mainly focused on cellular senescence mechanisms, such as telomerase activity, telomere shortening, oxidative stress, DNA damage, protein homeostasis imbalance, and mitochondrial dysfunction [9–11]. Of those mechanisms, oxidative stress was found to be an important factor that affected cellular senescence and organism aging.

Oxidative damage caused abnormal metabolism, increased the concentrations of ROS, activated DNA damage and protein homeostasis imbalance, and triggered direct or indirect regulation of senescence-related signaling pathways [12, 13]. One of the most important modulated senescent factors is the oxygen level present in the tissues [14]. The exposure of MSCs by long-term oxidative stress gives rise to cellular senescence and dysfunction [15]. Hypoxic conditions promoted oxidative stress, which may be implicated in stress-induced cellular senescence, and oxidative protein damage [16]. The MSC senescence model was exposed to oxidative stress using a hypoxia inducer or hydrogen peroxide to explore the influence of microRNAs or drugs on aging [17, 18]. However, these studies did not evaluate the overall protein level, and the potential underlying molecular mechanisms of senescence remain unclear. Tandem mass tag (TMT) quantitative analysis with liquid chromatography-tandem mass spectrometry (LC-MS/MS) is considered to be a sensitive and large-scale multiplexed proteomic approach, which facilitated the use of proteomic analyses in various areas of research [19]. Recent studies have shown a detailed description of proteomic profiling and pathway analysis related to aging in monkeys and rabbits, which contributed to further mechanistic studies and marker selection [20, 21]. Moreover, there are few studies exploring the changes in proteomic profiles in senescent cells using TMT-based LC-MS/MS. In this study, we aimed to determine the key differentially expressed proteins in senescent MSCs using TMT and parallel reaction monitoring (PRM) proteomic analyses. We also sought to explore the molecular mechanisms of oxidative stress-induced senescence.

## 2. Materials and Methods

### 2.1. Schematic of Proteomic Analysis in Senescent hBMSCs

**Figure 1:**
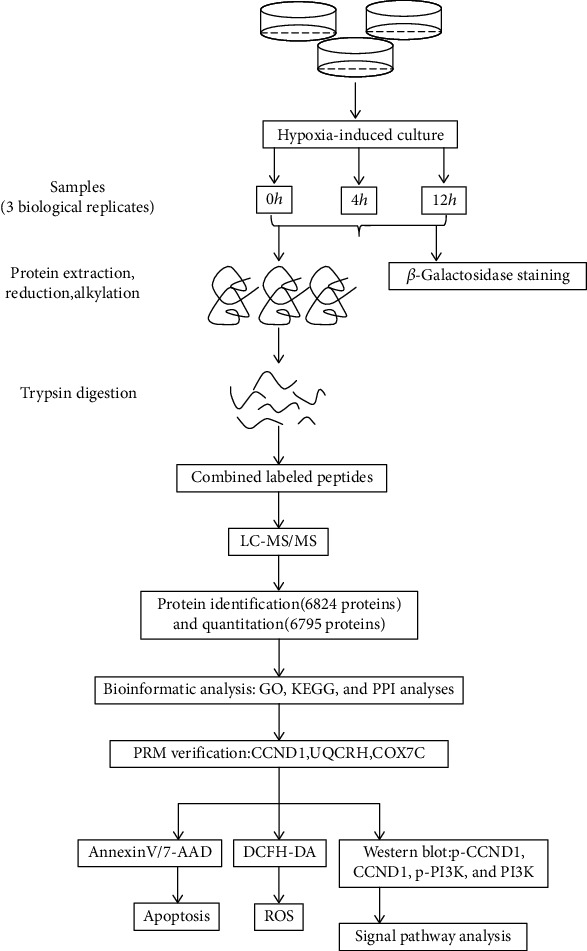
The complete experimental process. Hypoxia in hBMSCs was induced for 0, 4, and 12 hours, and cellular senescence was evaluated by senescence-associated *β*-galactosidase (SA-*β*-gal) staining. TMT quantitative proteomic analysis with LC-MS/MS analysis was performed to identify and quantify proteins. The general characterization of enriched proteins was performed by GO, KEGG, and PPI network analyses. PRM analysis was used to validate the candidate proteins (CCND1, UQCRH, and COX7C) with changes in expressions. Cell apoptosis was evaluated using Annexin V/7-AAD staining by flow cytometry. ROS was detected by the fluorescent probe DCFH-DA. Verifications of signaling pathways were evaluated by western blotting.

The workflow and characteristics of the proteomic analyses in the hypoxia-induced senescent hBMSCs are shown in Figure 1.

### 2.2. Cell Culture

Human bone marrow mesenchymal stem cells (hBMSCs) from three different donors were purchased from Cyagen Biotechnology Co., Ltd. (Art. No. HUXMA-0100, China). Research suggests that MSCs express specific surface markers like clusters of differentiation CD105, CD29, CD73, and CD44 and lack the expression of CD34, CD45, CD11b, and CD117 [22, 23]. Cells at passage 3 were identified, and the expression of CD105 > 85%, CD29 > 99%, CD73 > 99%, CD34 < 1%, CD45 < 1%, CD11b < 1%, CD117 < 1%, and CD44 > 99% was verified by flow cytometry [24]. The hBMSCs can differentiate into adipogenic or osteoblastic cells.

The hBMSCs were cultured in the Human Umbilical Cord MSCs Basal Medium (Cat. No. HUXUC-0311-440, Cyagen Biosciences, USA) with 10% Fetal Bovine Serum (Cat. No. HUXUC-05001-50, Cyagen Biosciences, USA). Cells were naturally passaged, and the growth of every passage was observed. When cells were 80–90% confluent, they were passaged 1 : 3. Cells were not passaged to below 40% confluence to prevent differentiation. Cells growing poorly or at passage 10 were discarded. Cells at passages 3 to 5 were digested with 0.25% trypsin-EDTA (Gibco, USA) and seeded into 6-well plates. A culture medium volume of 2 mL/well was added to the 6-well plates. When cells were 70–80% confluent, the complete medium was replaced with a fresh serum-free medium and cultured under hypoxic conditions for 0, 4, or 12 hours at 37°C in 1% O_2_, 5% CO_2_, and 94% N_2_ [25, 26]. Cells were collected at 0, 4, or 12 hours of hypoxia induction. Experiments were performed in three hBMSC cultures from three different donors.

### 2.3. *β*-Galactosidase Analysis

The cells were stained following the manual of the Senescence *β*-Galactosidase Staining Kit (Cell Signaling Technology, 9860S). The hBMSCs with different treatments were cultured in 6-well plates. They were washed once with phosphate-buffered saline (PBS), and a fixative solution was added at room temperature for 10 to 15 minutes. Cells were then washed twice with PBS, and SA-*β*-gal staining solution was added for overnight incubation at 37°C. Cells were then observed under an optical microscope. SA-*β*-gal-positive cells were stained blue and imaged in five random fields, and a total of at least 100 cells were counted in each sample [27]. The percentage of senescent hBMSCs was calculated based on the ratio of positive hBMSCs from five different fields. Experiments were performed in three hBMSC cultures from three different donors.

### 2.4. Preparation of Protein Samples

The experimental cells were divided into three groups: 0, 4, and 12 hours of hypoxia treatment. An SDS-dithiothreitol buffer (4% sodium dodecyl sulfate (SDS), pH 7.6, 1 mM dithiothreitol (DTT), and 100 mM Tris-HCl) was used for sample lysis and protein extraction. Protein quantification was performed using the Bicinchoninic Acid (BCA) Protein Assay Kit (Bio-Rad, USA). 100 *μ*g protein from each sample was trypsin-digested according to filter-aided sample preparation (FASP) [28]. Peptides were desalted, lyophilized, redissolved in 40 *μ*L of 0.1% (*v*/*v*) formic acid, and quantified (OD280). A total of 60 *μ*g of peptides was taken from each sample and labeled according to the manual of the Thermo TMT labeling kit. The labeled peptides were mixed and graded by the AKTA Purifier 100 (GE, USA). The peptide mixture was acidified with buffer A (pH 3.0, 10 mM KH_2_PO_4_ in 25% of ACN). The sample was loaded from the injector to a 4.6 × 100 mm column (5 *μ*m, 200 Å, PolyLC Inc., Maryland, USA) for separation. The peptides were eluted at a flow rate of 1 mL/min using a gradient of 0% buffer B (pH 3.0, 500 mM KCl, and 10 mM KH_2_PO_4_ in 25% of ACN) for 25 min, 0–10% buffer B for 25–32 min, 10–20% buffer B for 32–42 min, 20–45% buffer B for 42–47 min, 45–100% buffer B for 47–52 min, and 100% buffer B for 52–60 min. Buffer B was reset to 0% after 60 min. During the elution process, the absorbance at 214 nm was measured and fractions were collected every 1 min. The collected fractions were desalted on C18 cartridges after freeze-drying [29].

### 2.5. LC-MS/MS Analysis

Ultraperformance liquid chromatography-mass spectrometry (UPLC-MS) is the most frequently used analytical platform in the untargeted metabolomic study [30]. A Q Exactive mass spectrometer can significantly boost the number of protein analyses and become a proteomic analysis workhorse in many laboratories [31]. UPLC combined with a Q Exactive mass spectrometer has been widely applied to obtain insight into many biological events, especially in detecting the global abundance of proteins [32].

Samples were separated by high-performance liquid chromatography (HPLC) with a nanoliter flow rate and easy nanoliquid chromatography (NLC) and analyzed by a Q Exactive mass spectrometer (Thermo Scientific, USA). The positive ion mode was performed by MS. MS data were acquired via a data-dependent top 10 method, choosing the most abundant precursor ions (300–1800 *m*/*z*). The resolution of the first-order mass spectrometry was 70,000 at 200 *m*/*z*, the automatic gain control (AGC) target was set to 1*E*6, the maximum IT was 50 ms, and the dynamic exclusion duration was 60.0 s. Twenty mass spectrogram 2 (MS2) scans were collected after each full scan. Higher-energy collisional dissociation (HCD) was used as the MS2 activation type, and the resolution spectrum for HCD was set to 17,500 at 200 *m*/*z*. The normalized collision energy was 30 eV, and the underfill was 0.1%. The instrument was run with the peptide recognition mode enabled [33].

### 2.6. Bioinformatic analysis [34]

#### 2.6.1. Gene Ontology (GO) Annotation

To analyze the difference of protein expression between groups, a fold change (FC) of >1.2 or <0.83 and a *p* value of <0.05 were used to identify up- and downregulated proteins. NCBI Basic Local Alignment Search Tool (BLAST) + client software (NCBI-blast-2.2.28+-win32.exe) and InterProScan [35] were used to find homolog sequences for the selected differentially expressed proteins. The software program, Blast2GO [36], was used to annotate the target protein. The process could be summarized as BLAST, mapping, annotation, and annotation augmentation using InterProScan. The results of GO annotations were plotted using R scripts, a flexible statistical analysis toolkit by writing functions and scripts [37]. Enrichment of proteins in the gene ontology for biological processes (BP), molecular functions (MF), and cellular components (CC) were obtained [38].

#### 2.6.2. Kyoto Encyclopedia of Genes and Genomes (KEGG) Annotation

Following the annotation steps, KAAS (KEGG automatic annotation server) software was used to BLAST the differentially expressed proteins (http://geneontology.org/), which were then mapped to pathways in KEGG.

#### 2.6.3. Protein-Protein Interaction Analysis

The protein-protein interaction (PPI) information of the target proteins was searched on the IntAct database (https://www.ebi.ac.uk/intact/main.xhtml) or using STRING software (https://string-db.org/). The interaction network was generated and analyzed using Cytoscape software (https://www.cytoscape.org/, version 3.2.1) [39], and clusters of core PPI networks were identified with the Cytoscape plugin MCODE (https://apps.cytoscape.org/apps/mcode) [40]. The most significant modules were identified with a cutoff MCODE score of >5.

### 2.7. Target Analysis by Parallel Reaction Monitoring (PRM)

To further check the results of the TMT analysis, LC-PRM/MS analysis was applied as additional quantification, had highly specific spectra, and was available to confirm the identity of peptides. Briefly, the TMT protocol was used for peptide preparation. The stable isotope peptide was added to each sample and used as a standard internal reference [41, 42]. According to KEGG and PPI results, three interesting proteins, cyclin D1 (CCND1), ubiquinol cytochrome c reductase hinge (UQCRH), and cytochrome c oxidase subunit 7C (COX7C), were selected for targeted quantification and verification among all of the differentially expressed proteins. We detected the selected proteins by PRM after establishing the preexperimental method and determining them to be reliable and stable. The PRM results were analyzed with Skyline quantitative analysis [43]. The expression of target proteins was obtained in the sample with the corrected internal standard peptide signal [44].

### 2.8. Verification of Signaling Pathways

#### 2.8.1. Cell Culture and Treatment

The hBMSCs of passages 3 to 5 (P3-P5) were inoculated into 6-well plates (about 2∗10^5^ cells per well). Cells were divided into four groups: the control group (0-hour hypoxia-induced group), the 12-hour hypoxia-induced group, the 740 Y-P (a phosphoinositide 3-kinase (PI3K) activator, 20 *μ*M, TargetMol, USA)+12-hour hypoxia-induced group, and the LY294002 (a PI3K inhibitor, 10 *μ*M, Sigma, USA)+12-hour hypoxia-induced group. According to the manual of the kit, cells were pretreated with 740 Y-P or LY294002 for 30 min prior to hypoxia induction [45].

#### 2.8.2. Western Blot Analysis [46]

To confirm that CCND1 changes have close relationships with cell senescence and changes to the PI3K-dependent signaling pathway, we checked the protein expression of phospho-cyclin D1 (p-CCND1), CCND1, phospho-PI3-kinase (p-PI3K), and PI3K. After 0 or 12 hours of hypoxia induction, cells were washed three times with cold PBS and lysed with an appropriate amount of the radioimmunoprecipitation assay (RIPA) lysis buffer (Millipore, USA) on ice for 30 min. Then, cells were scraped, and the lysates were collected and centrifuged at 12,000 rpm for 15 min at 4°C. The supernatants were carefully aspirated and stored at -80°C. A phosphatase inhibitor cocktail was added before proteolysis. Protein concentrations were determined using a BCA kit (Thermo, USA) according to the manufacturer's instructions. The total amount of protein was adjusted to 15 *μ*g for consistency. Protein samples and the loading buffer were mixed at a ratio of 4 : 1. Then, proteins were separated by SDS-PAGE and transferred to polyvinylidene fluoride (PVDF) membranes. Subsequently, the PVDF membranes were blocked with 5% BSA at room temperature for 1 hour. Membranes were then incubated with primary antibodies against p-CCND1 (CST, USA, rabbit monoclonal antibody, 1 : 1000 dilution, 36 kDa), CCND1 (CST, USA, rabbit monoclonal antibody, 1 : 1000 dilution, 36 kDa), p-PI3K (CST, USA, rabbit antibody, 1 : 1000 dilution, 110 kDa), PI3K (CST, USA, rabbit monoclonal antibody, 1 : 1000 dilution, 110 kDa), and GAPDH (CST, USA, rabbit antibody, 1 : 5000 dilution, 37 kDa) overnight at 4°C. GAPDH was used as an internal control. The next day, membranes were washed three times with Tris Buffered Saline with 0.1% Tween 20 (TBST) and incubated with the anti-rabbit HRP secondary antibody (Jackson ImmunoResearch, USA, 1 : 5000) for 1 hour at room temperature. Membranes were analyzed using an ECL detection kit (GeneCopoeia, USA). The densitometry of protein bands was quantified by ImageJ software [47]. Experiments were performed in three hBMSC cultures from three different donors.

#### 2.8.3. Annexin V/7-AAD Staining for Assessing Apoptosis

In healthy cells, phosphatidylserine (PS) was only distributed in the inner leaflet of the lipid bilayer; however, in the early stage of apoptosis, PS on the cell membrane traversed from the inner leaflet to the outer leaflet. Annexin V can bind to PS and is used as an indicator of early apoptosis [48]. The 7-aminoactinomycin D (7-AAD) can bind to DNA and is a fluorescent probe used to detect late apoptotic and dead cells [49]. Therefore, the combination of Annexin V and 7-AAD can distinguish cells in different apoptotic stages. For the analysis of apoptosis, cells were double-stained using 5 *μ*L Annexin V and 10 *μ*L 7-AAD (BD Pharmingen, USA) and then analyzed by flow cytometry [50]. Experiments were performed in three hBMSC cultures from three different donors.

#### 2.8.4. Reactive Oxygen Species (ROS) Assay

2,7-Dichlorodihydrofluorescein acetoacetic acid (DCFH-DA) is a cell-permeable probe that detects intracellular ROS. About 2∗10^5^ cells were incubated in static with H2DCF-DA (5 *μ*M, MCE, USA) in the dark for 30 min at 37°C; then, cells were harvested with a 0.05% trypsin-EDTA solution, resuspended in a fresh medium, and detected by flow cytometry [51]. Mean fluorescence intensity (MFI) in cells was calculated. Experiments were performed in three hBMSC cultures from three different donors.

### 2.9. Statistical Analysis

SPSS 22.0 statistical software was used for data analysis. All data were expressed as the mean ± SD. Groups were compared using *t*-tests, and *p* < 0.05 was considered statistically significant.

## 3. Results

### 3.1. Senescence Detection of hBMSCs after Hypoxia Treatment

**Figure 2:**
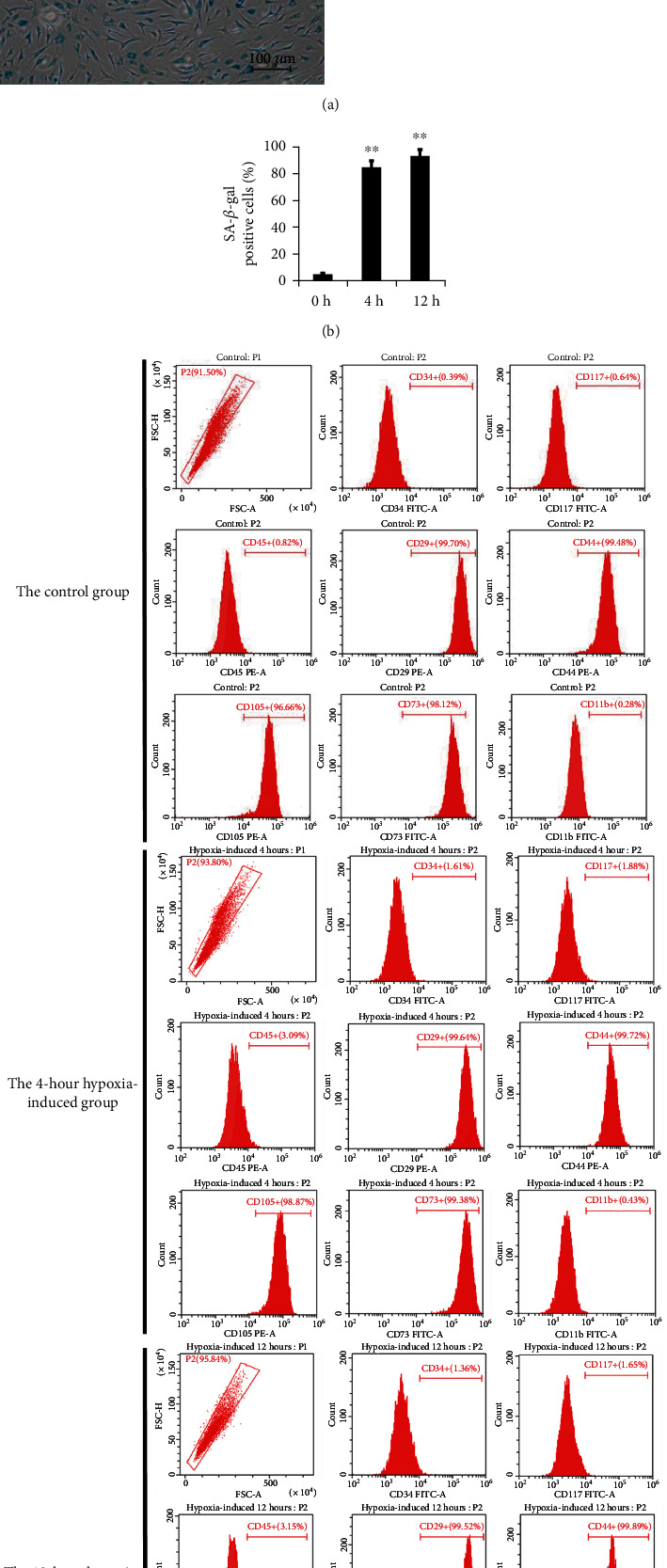
Hypoxia-induced cell senescence at different time points and surface marker profiles. (a) Representative images of senescent hBMSCs using SA-*β*-gal staining. Blue: SA-*β*-gal-positive cells. Scale bar = 100 *μ*m. (b) Quantification of SA-*β*-gal-positive cells. The percentage of SA-*β*-gal-positive cells was significantly increased after hypoxia treatment, compared to the control group. 0 h, 4 h, and 12 h: time of hypoxia treatment. Experiments were performed in three hBMSC cultures from three different donors. Mean ± SD. ^∗∗^*p* < 0.01 vs. the 0 h control group. (c) The expressions of surface markers such as CD34, CD117, CD45, CD29, CD44, CD105, CD73, and CD11b were not significantly different between the hypoxia-induced group and the control group.

Hypoxic conditions (1% O_2_ and 94% N_2_) were used to induce hBMSC senescence. The percentage of SA-*β*-gal-positive cells was significantly increased after hypoxia treatment, compared to the control group. The expressions of CD105, CD29, CD73, CD34, CD45, CD11b, CD117, and CD44 were verified by flow cytometry. There was no significant difference in surface markers between the 12-hour hypoxia-induced group and the control group (Figure 2).

### 3.2. Proteomic Analysis Based on TMT

**Figure 3:**
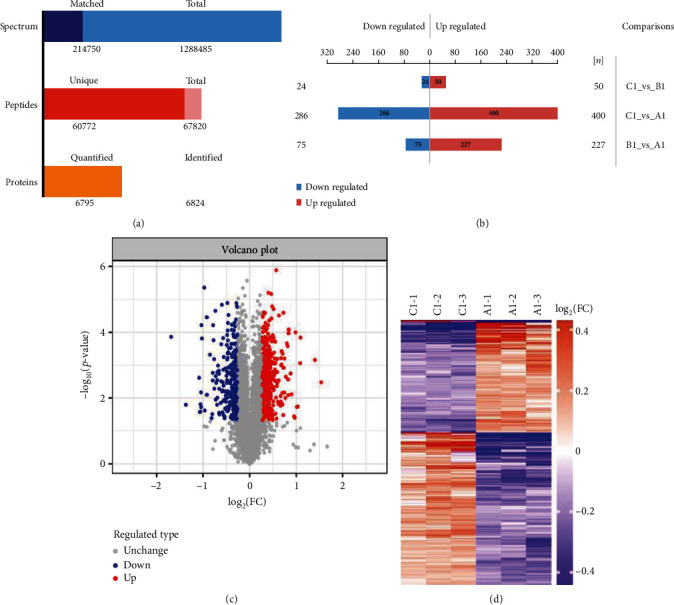
Profiling of differentially expressed proteins between the hypoxia-induced group and the control group. (a) Statistical histogram of protein identification and quantification results by TMT. A total of 6824 proteins were identified, of which 6795 were quantified. (b) The up- (red) or downregulated (blue) proteins were displayed in different groups. 74 significantly differentially expressed proteins were identified, of which 50 were upregulated and 24 were downregulated after hypoxia induction for 12 hours (group C1), compared to hypoxia induction for 4 hours (group B1). 686 significantly differentially expressed proteins were identified, of which 400 were upregulated and 286 were downregulated after hypoxia induction for 12 hours (group C1), compared to the control group (group A1). 302 significantly differentially expressed proteins were identified, of which 227 were upregulated and 75 were downregulated after hypoxia induction for 4 hours (group B1), compared to the control group (group A1). (c) Volcano plot showing the up- (red) or downregulated (purple) proteins between the 12-hour hypoxia-induced group (group C1) and the control group (group A1). (d) The hierarchical clustering heat map of differentially expressed proteins between the 12-hour hypoxia-induced group (group C1) and the control group (group A1). Each column represents a set of samples, while each row represents a protein in the figure. The red represents significantly higher proteins, while purple represents significantly lower proteins in the heat map; the gray part means quantitative information without protein.

From the LC-MS/MS analysis, 1,288,485 spectra were obtained. After filtering to remove low-scoring spectra, there were 214,750 spectra that could be matched to 67,820 peptides, of which 60,772 were unique. A total of 6824 proteins were identified, of which 6795 were quantified (Figure 3(a)). To analyze the difference of protein expression between groups, a fold change (FC) of >1.2 or <0.83 and a *p* value of <0.05 were used to identify up- and downregulated proteins. Based on these criteria, 686 significantly differentially expressed proteins were identified, of which 400 were upregulated and 286 were downregulated after hypoxia induction for 12 hours (compared to the control group (Figure 3(b))). Volcano plots and hierarchical clustering heat maps showed the protein expression changes between the hypoxia group (12 h) and the control group (Figures 3(c) and 3(d)).

### 3.3. Gene Ontology Analysis of the Differentially Expressed Proteins

**Figure 4:**
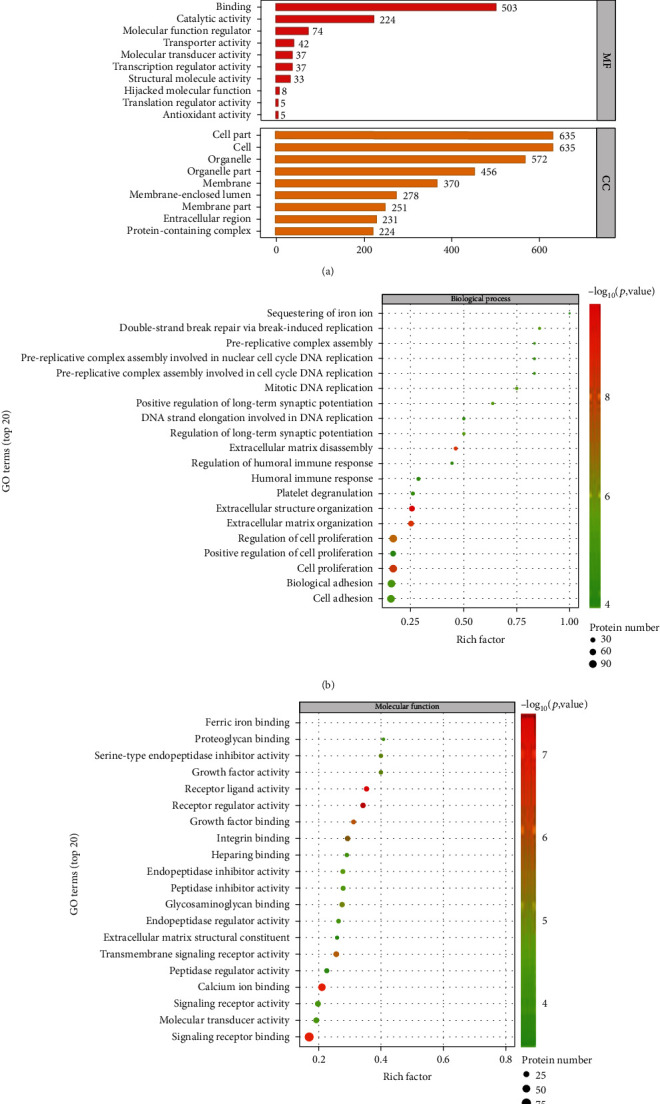
Profiling of differentially expressed proteins using GO analysis. (a) GO annotated statistics of differentially expressed proteins. The gene ontology categories of differentially expressed proteins based on biological processes (BP), molecular functions (MF), and cellular components (CC) between the 12-hour hypoxia-induced group and the control group. (b) As for BP, the upregulated (red) differentially expressed proteins were involved in extracellular structure organization, extracellular matrix organization, extracellular matrix disassembly regulation, cell proliferation, and regulation of cell proliferation. The downregulated (green) proteins were mainly enriched in the positive regulation of cell proliferation, biological adhesion, cell adhesion, platelet degranulation, and humoral immune response categories. (c) In terms of MF, the upregulated (red) proteins were mainly annotated in the receptor-ligand activity, receptor regulator activity, signaling receptor binding, calcium ion binding, and growth factor binding categories. Downregulated (green) proteins were mainly enriched in the peptidase regulator activity, extracellular matrix structural constituent, molecular transducer activity, and signaling receptor activity categories.

To understand the function, location, and biological pathway of 686 significantly differentially expressed proteins, we annotated them based on biological processes (BP), molecular functions (MF), and cellular components (CC) (Figure 4(a)) and performed GO enrichment analyses. Compared to the control group, the results of the hypoxia group (12 h) showed that the most significantly enriched biological processes for the upregulated proteins were extracellular structure organization, extracellular matrix organization, extracellular matrix disassembly regulation, cell proliferation, and regulation of cell proliferation. The downregulated proteins were mainly enriched in the positive regulation of cell proliferation, biological adhesion, cell adhesion, platelet degranulation, and humoral immune response categories (Figure 4(b)). In terms of molecular functions, the upregulated proteins were mainly annotated in the receptor-ligand activity, receptor regulator activity, signaling receptor binding, calcium ion binding, and growth factor binding categories. The downregulated proteins were mainly enriched in the peptidase regulator activity, extracellular matrix structural constituent, molecular transducer activity, and signaling receptor activity categories (Figure 4(c)).

### 3.4. KEGG Pathway Analysis of Senescence-Related Proteins of hBMSCs

**Figure 5:**
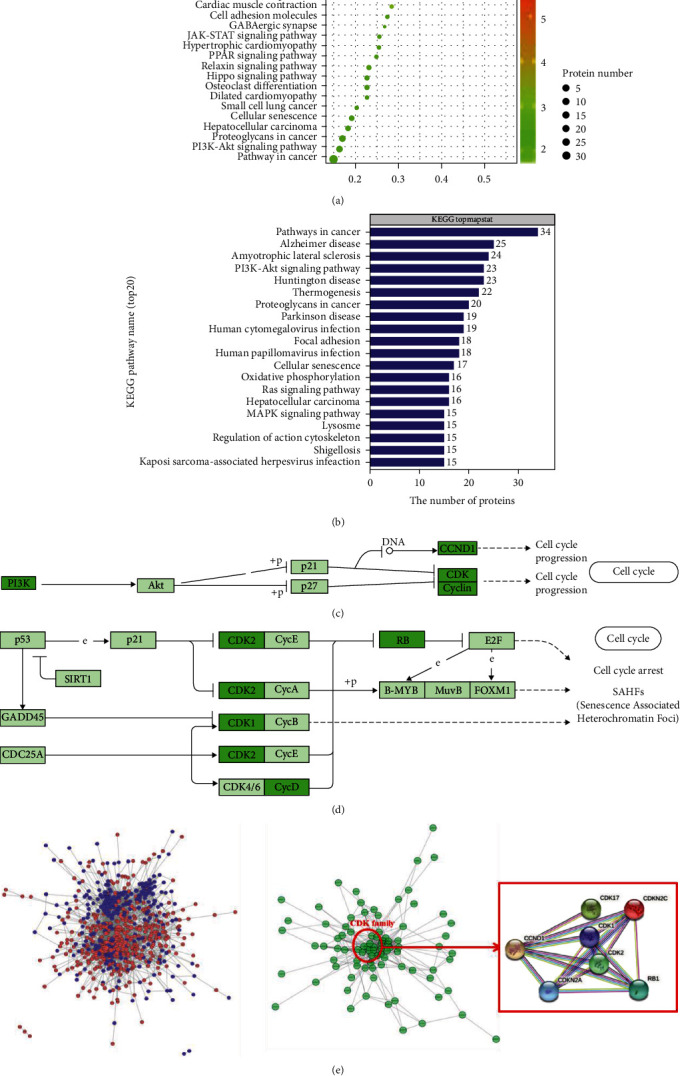
KEGG pathway analysis of hypoxia-induced hBMSCs. (a) KEGG pathway enrichment analysis of differentially expressed proteins from cells treated with or without hypoxia. The upregulated (red) proteins were mainly involved in cytokine-cytokine receptor interactions and complement and coagulation cascades, whereas the downregulated (green) proteins were mainly involved in cancer pathways, the PI3K-Akt signaling pathway, proteoglycans in cancer, hepatocellular carcinoma, and cellular senescence. (b) KEGG pathway annotation statistics of differentially expressed proteins (top 20). The signaling pathway annotations of differentially expressed proteins were critically related to pathways in cancer (34 proteins), Alzheimer disease (25 proteins), amyotrophic lateral sclerosis (24 proteins), the PI3K-Akt signaling pathway (23 proteins), and Huntington disease (23 proteins). (c) The role of the CDK cluster and CCND1 in the PI3K signaling pathway. (d) The role of the CDK cluster in the p53 signaling pathway. (e) PPI network of differentially expressed proteins in the 12-hour hypoxia-induced group. PPI analyses indicated that CDK1, CDK2, and CCND1 were important nodes. STRING network bonds. Blue: from curated databases; rose pink: experimentally determined; green: gene neighborhood; red: gene fusions; navy blue: gene co-occurrence; yellow: text mining; black: coexpression; purple: protein homology.

Compared to the control group, the results of KEGG pathway enrichment analyses showed that the upregulated proteins were mainly involved in cytokine-cytokine receptor interactions and complement and coagulation cascades, whereas the downregulated proteins were mainly involved in cancer pathways, the PI3K-Akt signaling pathway, proteoglycans in cancer, hepatocellular carcinoma, and cellular senescence (Figure 5(a)). Meanwhile, KEGG pathway annotation statistics of differentially expressed proteins were critically related to pathways in cancer (34 proteins), Alzheimer disease (25 proteins), amyotrophic lateral sclerosis (24 proteins), the PI3K-Akt signaling pathway (23 proteins), and Huntington disease (23 proteins) (Figure 5(b)). The PI3K-Akt and p53 signaling pathways are important senescence regulation pathways. Many proteins in the PI3K-Akt and p53 signaling pathways were influenced by hypoxia treatment for 12 hours (Figures 5(c) and 5(d)).

To better reveal the interaction among hypoxia-induced senescence, we performed PPI network analyses using Cytoscape software based on the STRING database. In the complex signaling cascades, the clusters were critically related to cancer, Alzheimer disease, amyotrophic lateral sclerosis, and Huntington disease. And most of these diseases are associated with cell senescence [52–55]. Combined with the TMT and KEGG results, we focused on a group of close interactions of cell cycle-related proteins (CDK1, CDK2, and CCND1), discovered in the PPI network, suggesting that protein synthesis was under the regulation of tight junctions during senescence (Figure 5(d)). PPI analyses indicated that CDK1, CDK2, and CCND1 were important nodes (Table S3). Furthermore, most of those proteins were downregulated in the 12-hour hypoxia-induced group, compared to the control group.

### 3.5. PRM-Validated Protein Expression Levels

**Figure 6:**
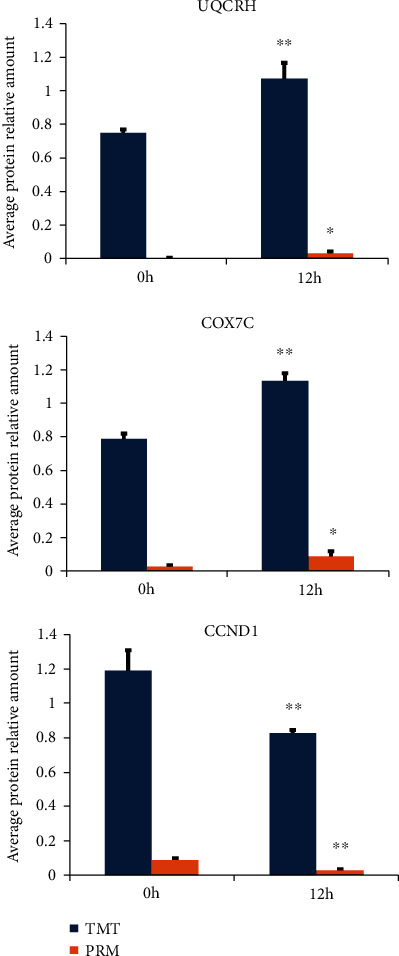
Expressions of UQCRH, COX7C, and CCND1 were analyzed by TMT and PRM. When hBMSCs were hypoxia-induced for 12 hours, expressions of UQCRH and COX7C were upregulated, while that of CCND1 was downregulated in TMT and PRM analyses. Mean ± SD. ^∗^*p* < 0.05 and ^∗∗^*p* < 0.01 vs. the 0 h control group. Experiments were performed in three hBMSC cultures from three different donors.

CCND1, COX7C, and UQCRH are important in promoting oxidative stress and inducing cell senescence [56–59]. To confirm the reliability of the quantitative proteomic analyses, combined with KEGG and PPI results, the candidate proteins UQCRH, COX7C, and CCND1 from the hypoxia-induced group (12 h) were evaluated by PRM analyses. They all had significant peptide quantitative information in every sample. UQCRH and COX7C were upregulated, while CCND1 was downregulated; all of the changes were significant when hBMSCs were hypoxia-induced for 12 hours (*p* < 0.05) in both the TMT and PRM analyses (Figure 6). The results of the relative quantification demonstrated that the target proteins displayed similar trends between the TMT and PRM analyses, thus supporting the reliability of the proteomic data.

### 3.6. Activation of the PI3K Could Antagonize the Effect of CCND1 in the Hypoxia-Induced Senescence of hBMSCs

**Figure 7:**
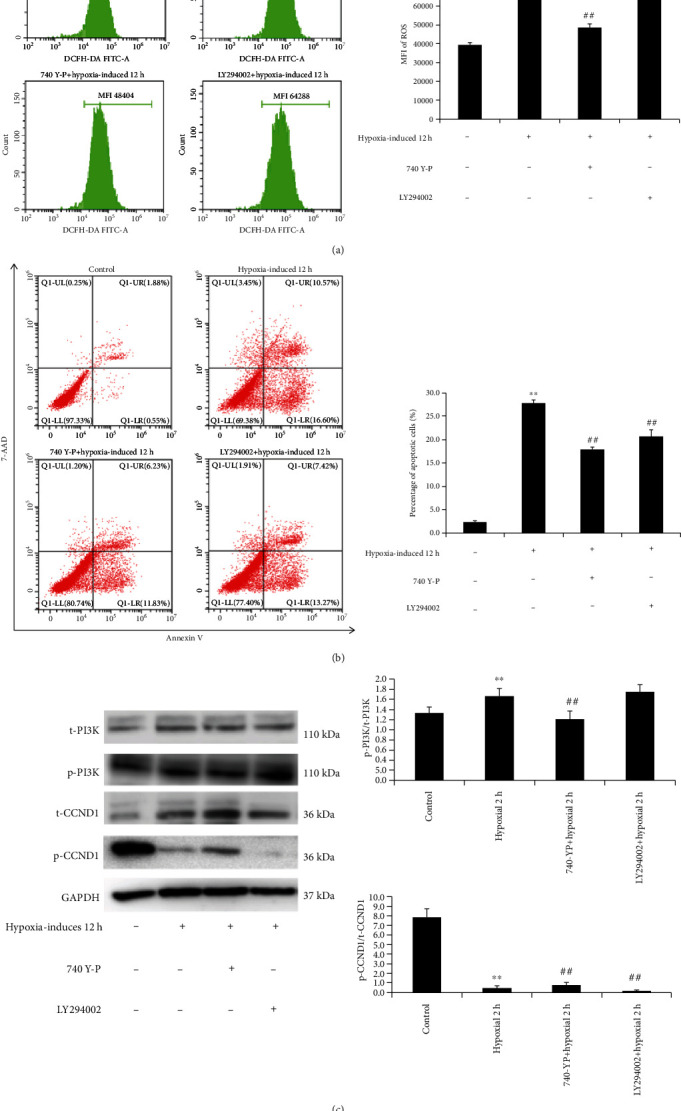
The activation of the PI3K antagonizes the effects of CCND1 on reactive oxygen species (ROS) production and apoptosis in hypoxia-induced senescent hBMSCs. (a) Hypoxia promotes ROS generation, which was reduced by pretreatment with 740 Y-P. Fluorescein isothiocyanate- (FITC-) labeled 2,7-dichlorodihydrofluorescein acetoacetic acid (DCFH-DA) was used to detect intracellular ROS by flow cytometry, and mean fluorescence intensity (MFI) in cells was calculated. (b) The percentage of apoptotic cells was measured by Annexin V/7-AAD staining. Cells apoptosis was significantly decreased by 740 Y-P treatment compared to the hypoxia-induced group. (c) Total PI3K (t-PI3K), phospho-PI3K (p-PI3K), total CCND1 (t-CCND1), and phospho-CCND1 (p-CCND1) protein expressions were analyzed by western blotting and quantified by densitometry. The p-PI3K/t-PI3K ratio in hypoxia-induced senescent cells was significantly higher than that of the control group; however, the p-CCND1/t-CCND1 ratio was decreased after hypoxia induction. Mean ± SD. ^∗∗^*p* < 0.01 vs. the 0 h control group. ^##^*p* < 0.01 vs. the 12-hour hypoxia-induced group. Western blot bands of p-PI3K (110 kDa), t-PI3K (110 kDa), p-CCND1 (36 kDa), t-CCND1 (36 kDa), and GAPDH (37 kDa). Experiments were performed in three hBMSC cultures from three different donors.

To further confirm that proteomic changes are related to cell senescence and apoptosis, ROS and cell apoptosis were detected in hypoxia-induced hBMSCs. As shown in Figures 7(a) and 7(b), continuous exposure to hypoxic conditions induced noticeable ROS generation and apoptosis in hBMSCs, compared to the control group, by suppressing the expression of CCND1 and the activation of the PI3K-dependent signaling pathway. We treated hypoxic hBMSCs with the PI3K activator, 740 Y-P, to analyze the change of CCND1 expression. The activation mechanism of PI3K involves relief of autoinhibition through binding of PI3K to phosphorylated tyrosines on a cell surface receptor. Generally, the activation of phosphorylation is the increase of the percentage of phosphorylated protein in the total protein. Therefore, we analyzed the level of phosphorylated protein and total protein in the PI3K signaling pathway and compared the phosphorylated protein/total protein ratio between groups [60]. The results showed that the expression of p-CCND1 was downregulated, while treatment with 740 Y-P increased p-CCND1 and treatment with LY294002 inhibited the phosphorylation of CCND1. Thus, 740 Y-P treatment decreased apoptosis and ROS levels in the hypoxia-induced group. Collectively, hypoxia-induced hBMSCs inhibited CCND1 expression and promoted ROS production and apoptosis, which were prevented by treatment with the PI3K agonist, 740 Y-P (Figure 7(c)).

## 4. Discussion

Cell senescence, as a hallmark of aging, caused various pathological phenotypes [61]. There are two main types of cellular senescence, including replicative senescence and pathological senescence [62]. Oxidative stress was found to be an important factor that affected cellular senescence and organism aging [63]. Hypoxia-induced senescence is a pathological senescence model of oxidative stress. In this study, hBMSCs were hypoxia-induced for 0, 4, and 12 hours, and the percentages of SA-*β*-gal-positive cells in 4-hour and 12-hour hypoxia-induced groups were more obviously increased than that of the control group. Almost all the cells were SA-*β*-gal-positive cells (93%) after 12 hours of hypoxia treatment (Figure 2). Subsequently, proteomic analyses based on TMT identified 686 proteins, of which 400 proteins were upregulated and 286 were downregulated in the hypoxia-induced group (12 h) (Figure 3). 302 significantly differentially expressed proteins were identified, of which 227 were upregulated and 75 were downregulated after hypoxia induction for 4 hours (Figure S2). The number of differentially expressed proteins in the 12-hour hypoxia-induced group was more than that in the 4-hour hypoxia-induced group. Therefore, we focus on the analysis of hypoxia induction for 12 hours as a key point for the follow-up study. Meanwhile, the collective results suggested that dramatic changes in gene expression occur during hBMSC senescence. Cellular senescence is an adaptive cellular process that occurs in response to noxious stimuli and is activated by upregulated proteins, including suppressors [64].

GO enrichment analyses identified that downregulated proteins were mainly enriched in the positive regulation of cell proliferation and integral components of the membrane categories (Figure 4). Those data also suggested that cell proliferation arrest was a hallmark of hypoxia-induced hBMSC senescence.

At present, the molecular mechanisms of stem cell senescence are unclear. The PI3K/Akt pathway is considered to be one of the most important signaling pathways for regulating cellular senescence. The PI3K/Akt signal was inhibited after long-term culture of MSCs *in vitro* [65]. The p53 signaling pathway is also important for regulating cellular senescence [66]. Additionally, the AMPK/PI3K signaling pathway regulates senescence also by regulating autophagy and cell proliferation. This pathway has been reported to extend longevity in different species through the regulation of physiological responses [67]. Therefore, the AMPK/PI3K and p53/p21 signaling pathways are important mechanisms of senescence. Our results also demonstrated that differentially expressed proteins in hypoxia-induced senescence are associated with PI3K and p53 signaling pathways.

Within PPI networks, highly aggregated proteins may have the same or similar functions. The greater the protein connectivity, the more important it is in maintaining the balance and stability of cell biology systems. Recent studies have confirmed that cyclin-dependent kinase 2 (CDK2) is a crucial protein in the p53 signaling pathway [68]. CDK2 is involved in the control of the cell cycle and is activated by interactions with cyclin E during the early stages of DNA synthesis to permit the G1-S transition and promote the transition from the S phase to mitosis.

When cells are damaged, p53 induces the expression of p21 by recognizing disabled telomeres. p53 then inactivates several cyclin-dependent kinases (CDKs), such as CDK2, CDK4, and CDK6, thus preventing retinoblastoma protein (Rb) phosphorylation, contributing to cell cycle arrest in the G1/S phase, and eventually causing cell senescence [69]. As our results indicated, CDK1 also participates in hypoxia-induced senescence and is involved in the p53 signaling pathway. CDK1 has more nodes than CDK2 when hBMSCs were hypoxia-induced for 12 hours. Thus, CDK1 is an important protein in cellular senescence. Senescent cells accumulate in tissues and may give rise to organ dysfunction and increase the risk of aging-associated diseases, such as cancer, cardiovascular disorders, and neurodegenerative diseases [57, 70–72]. CDK1 is involved in cell proliferation and is a predictive tumor marker [73]. Cyclin D1 (CCND1) is also an important node in the PPI network and activates CDK4 and CDK6, thereby leading to cell cycle arrest. CCND1 is involved in senescence and amplification, which may be key points associated with tumor proliferation and apoptosis [74]. Continuous exposure to hypoxia suppressed the expression of CCND1. Therefore, current research studies on the CDK family of proteins are mostly related to tumorous and neurodegenerative diseases but few in stem cells. In this study, we found that the expressions of CDK1, CDK2, and CCND1 were significantly downregulated in hypoxia-induced senescence of MSCs (Figure 5). These findings suggested that the CDK family of proteins was closely related to cell proliferation arrest and played a core regulatory role in oxidative stress-induced hBMSC senescence.

Tandem mass tag (TMT) proteomics is an *in vitro* labeling technique widely used in the analysis of differentially expressed proteins. Because TMT analysis is a large-scale data acquisition process, it is necessary to verify differently expressed proteins if we want to ensure the accuracy of proteomic results and conclusions. So we used flow cytometry and western blot analysis to verify the expression change of CD44 before and after hypoxia treatment in hBMSCs. It was worth noting that CD44 was upregulated in the PPI network (Table S1, S2, S3, S4) after hypoxia [75–109]. However, there was no significant difference in CD44 by flow cytometry (Figure 2(b)) and western blot analysis between the 12-hour hypoxia-induced group and the control group (Figure S1). Flow cytometry and western blot are more specific and accurate than large-scale data collection. Our results of this experiment showed that the expression of CD44 met the standard of BM-MSC purification. Moreover, CD44 was used as a specific marker for MSC differentiation [22]. CD44 is a cell adhesion molecule involved in cell-cell and cell-extracellular matrix communications [110]. Present studies of CD44 mainly focused on the correlation with tumors [111] but few with MSCs. In our study, CD44 was not found to be differentially expressed following western blot analysis and flow cytometry validation. The CD44 signaling pathway may not be involved in the mechanism of hypoxia-induced senescence of hBMSCs, suggesting that CD44 may not be stimulated by oxidative stress in hBMSCs. Moreover, UQCRH and COX7C related to mitochondrial dysfunction attracted our attention.

Mitochondrial dysfunction has been suggested as another main cause of aging [112, 113]. Previous studies revealed that UQCRH and COX7C are associated with mitochondrial dysfunction in cells experiencing oxidative damage. UQCRH and COX7C proteins modulate mitochondrial functions by reducing oxidative damage and imparting cellular defense strategies against oxidative stress in chronic diseases and tumors [57–59, 78]. They also play important roles in oxidative phosphorylation by participating in the Nrf2 and AMPK/PI3K signaling pathways [114]. UQCRH and COX7C were upregulated in our exploratory research (Figure 6), and it was revealed that those proteins are likely to play a role in the hypoxia-induced senescence of hBMSCs. However, the functions and molecular mechanisms of UQCRH and COX7C in cellular senescence need further investigation.

All of the results of this study suggested that oxidative damage triggered ROS production, mitochondrial dysfunction, and cell proliferation and eventually led to cellular senescence and apoptosis. According to the proteomic profiling results, the PI3K-dependent pathway may be an important signaling pathway to regulate cellular senescence. To confirm that proteomic changes have close relationships with cell senescence and changes to signaling pathways, we further treated hypoxia-induced hBMSCs with the PI3K activator, 740 Y-P. We found that hypoxia-induced hBMSCs exhibited reduced expression of CCND1 and promoted ROS production and apoptosis (Figure 7). These changes were reversed when cells were treated with the PI3K activator, 740 Y-P, thus indicating that such changes were exerted via CCND1 and the PI3K pathway. Furthermore, the levels of p-PI3K increased significantly in the hypoxia-induced group; however, there was no significant change in total PI3K.

In summary, our study was the first to comprehensively analyze proteomic changes in hBMSCs undergoing hypoxia-induced senescence. We identified 686 differentially expressed proteins, among which the major functions were annotated as “cellular process” and “binding.” The PI3K/Akt and p53 pathways exhibited the most notable changes among these signaling pathways. CDK1, CDK2, and CCND1 were also highlighted as important protein nodes regulating oxidative stress-induced cellular senescence. Oxidative stress inhibited CCND1 and promoted ROS production and apoptosis in senescent stem cells via the PI3K-dependent signaling pathway. Therefore, oxidative stress inhibits hBMSC proliferation, increases cellular senescence, and promotes cell apoptosis. The modulation of the expression and activity of the CDK family may be an ideal method to reduce oxidative stress in hBMSCs. Our research provides a better understanding of the molecular mechanisms of hypoxia on proteomic profiles changes of hBMSCs. The differentially expressed proteins act in precise molecular mechanisms that need further studies.

## Data Availability

The data used to support the findings of this study are available from the corresponding author upon request.

## Supplementary Materials

Supplementary Materials Table S1: top 15 upregulated differentially expressed proteins of senescence between the hypoxia-induced group and the control group. Table S2: top 15 downregulated differentially expressed proteins of senescence between the hypoxia-induced group and the control group. Table S3: notes of top 15 differentially expressed proteins in PPI network analyses between the hypoxia-induced group and the control group. Table S4: a list of 400 upregulated significantly differentially expressed proteins between the hypoxia-induced group and the control group. Table S5: a list of 286 downregulated significantly differentially expressed proteins between the hypoxia-induced group and the control group. Figure S1: CD44 protein expressions were analyzed by western blotting and quantified by densitometry. Figure S2: profiling of differentially expressed proteins between the 4-hour hypoxia-induced group and the control group. Figure S3: profiling of differentially expressed proteins using GO analysis between the 4-hour hypoxia-induced group and the control group. Figure S4: KEGG pathway analysis of hBMSCs between the 4-hour hypoxia-induced group and the control group.
