# Supplementary material for: Proteomic Analysis of Hypoxia-Induced Senescence of Human Bone Marrow Mesenchymal Stem Cells
Source: Stem Cells Int. 2021 Aug 27;2021:5555590. doi: 10.1155/2021/5555590 (PMC8416403; doi:10.1155/2021/5555590)
Supplement: Supplementary Materials — Table S1: top 15 upregulated differentially expressed proteins of senescence between the hypoxia-induced group and the control group. Table S2: top 15 downregulated differentially expressed proteins of senescence between the hypoxia-induced group and the control group. Table S3: notes of top 15 differentially expressed proteins in PPI network analyses between the hypoxia-induced group and the control group. Table S4: a list of 400 upregulated significantly differentially expressed proteins between the hypoxia-induced group and the control group. Table S5: a list of 286 downregulated significantly differentially expressed proteins between the hypoxia-induced group and the control group. Figure S1: CD44 protein expressions were analyzed by western blotting and quantified by densitometry. Figure S2: profiling of differentially expressed proteins between the 4-hour hypoxia-induced group and the control group. Figure S3: profiling of differentially expressed proteins using GO analysis between the 4-hour hypoxia-induced group and the control group. Figure S4: KEGG pathway analysis of hBMSCs between the 4-hour hypoxia-induced group and the control group. [file 5555590.f1.zip › 5555590.f1.pdf]

International Science Editing

www.internationalscienceediting.com

DATE: February 03, 2021

Compuscript Ltd
T/A International Science Editing
Bay K, Shannon Industrial Park West
Shannon, Co Clare
Ireland
Phone +353 61 472818 Fax +353 61 472688

To whom it may concern,

The paper “Proteomics analysis of hypoxia-induced senescence of human bone marrow mesenchymal stem cells” by Liping Mai was edited by International Science Editing. We were asked not to edit the references. Please contact us if you would like to view the edited paper.

Kindest regards,

David Cushley.
