# Supplementary material for: Proteomic Analysis of Hypoxia-Induced Senescence of Human Bone Marrow Mesenchymal Stem Cells
Source: Stem Cells Int. 2021 Aug 27;2021:5555590. doi: 10.1155/2021/5555590 (PMC8416403; doi:10.1155/2021/5555590)
Supplement: Supplementary Materials — Table S1: top 15 upregulated differentially expressed proteins of senescence between the hypoxia-induced group and the control group. Table S2: top 15 downregulated differentially expressed proteins of senescence between the hypoxia-induced group and the control group. Table S3: notes of top 15 differentially expressed proteins in PPI network analyses between the hypoxia-induced group and the control group. Table S4: a list of 400 upregulated significantly differentially expressed proteins between the hypoxia-induced group and the control group. Table S5: a list of 286 downregulated significantly differentially expressed proteins between the hypoxia-induced group and the control group. Figure S1: CD44 protein expressions were analyzed by western blotting and quantified by densitometry. Figure S2: profiling of differentially expressed proteins between the 4-hour hypoxia-induced group and the control group. Figure S3: profiling of differentially expressed proteins using GO analysis between the 4-hour hypoxia-induced group and the control group. Figure S4: KEGG pathway analysis of hBMSCs between the 4-hour hypoxia-induced group and the control group. [file 5555590.f1.zip › 5555590.f2.docx]

**Supplement1**

**Table S1 Top 15 up-regulated differentially expressed proteins of senescence between the hypoxia-treatment group and the control group.**

| **Accession** | **Protein Name** | **Gene Name** | **Fold change** |
| --- | --- | --- | --- |
| Q13501 | Sequestosome-1^75,76^ | SQSTM1 | 1.79 |
| P17936 | Insulin-like growth factor-binding protein 3^77^ | IGFBP3 | 1.68 |
| Q53EL6 | Programmed cell death protein 4^78,79^ | PDCD4 | 1.51 |
| Q13541 | Eukaryotic translation initiation factor 4E-binding protein 1^80^ | EIF4EBP1 | 1.47 |
| P07585 | Decorin^81^ | DCN | 1.45 |
| P63098 | Calcineurin subunit B type 1^82^ | PPP3R1 | 1.41 |
| P16035 | Metalloproteinase inhibitor 2^83^ | TIMP2 | 1.41 |
| P37173 | TGF-beta receptor type-2^84^ | TGFBR2 | 1.40 |
| P09619 | Platelet-derived growth factor receptor beta^85^ | PDGFRB | 1.39 |
| P01210 | Proenkephalin-A^86^ | PENK | 1.38 |
| Q9UKB1 | F-box/WD repeat-containing protein 11^87^ | FBXW11 | 1.37 |
| P08473 | Neprilysin^88^ | MME | 1.34 |
| P60602 | Reactive oxygen species modulator 1^89^ | ROMO1 | 1.27 |
| P27797 | Calreticulin^90^ | CALR | 1.26 |
| P04439 | HLA class I histocompatibility antigen, A alpha chain^91^ | HLA-A | 1.25 |

**Table S2 Top 15 down-regulated differentially expressed proteins of senescence between the hypoxia-treatment group and the control group.**

| **Accession** | **Protein Name** | **Gene Name** | **Fold change** |
| --- | --- | --- | --- |
| P04818 | Thymidylate synthase^92^ | TYMS | 0.58 |
| P06400 | Retinoblastoma-associated protein^93,94^ | RB1 | 0.62 |
| P47974 | mRNA decay activator protein ZFP36L2^95^ | ZFP36L2 | 0.65 |
| Q15493 | Regucalcin^96^ | RGN | 0.67 |
| P24385 | G1/S-specific cyclin-D1^97^ | CCND1 | 0.69 |
| P24941 | Cyclin-dependent kinase 2^98, 99^ | CDK2 | 0.70 |
| P07992 | DNA excision repair protein ERCC-1^100^ | ERCC1 | 0.72 |
| Q6IQ20 | N-acyl-phosphatidylethanolamine-hydrolyzingphospholipase D^101^ | NAPEPLD | 0.75 |
| P29279 | CCN family member 2^102^ | CCN2 | 0.76 |
| O14929 | Histone acetyltransferase type B catalytic subunit^103^ | HAT1 | 0.77 |
| O14807 | Ras-related protein M-Ras^104^ | MRAS | 0.78 |
| P06493 | Cyclin-dependent kinase 1^105,106^ | CDK1 | 0.79 |
| O43294 | Transforming growth factor beta-1-induced transcript 1 protein^107^ | TGFB1I1 | 0.82 |
| P36873 | Serine/threonine-protein phosphatase PP1-gamma catalytic subunit^108^ | PPP1CC | 0.82 |
| Q6XZF7 | Dynamin-binding protein^109^ | DNMBP | 0.83 |

**Table S3 Notes of top 15 differentially expressed proteins in PPI network analyses between the hypoxia-treatment group and the control group.**

| **Accession** | **Nodes** | **Protein Name** | **Gene Name** |
| --- | --- | --- | --- |
| P02768 | 105 | Serum albumin | ALB |
| P02751 | 96 | Fibronectin | FN1 |
| P0CG48 | 73 | Polyubiquitin-C | UBC |
| P60709 | 71 | Actin, cytoplasmic 1 | ACTB |
| P16070 | 63 | CD44 antigen | CD44 |
| P06493 | 62 | Cyclin-dependent kinase 1 | CDK1 |
| P24385 | 58 | G1/S-specific cyclin-D1 | CCND1 |
| P05067 | 58 | Amyloid-beta precursor protein | APP |
| P08253 | 55 | 72 kDa type IV collagenase | MMP2 |
| P07237 | 48 | Protein disulfide-isomerase | P4HB |
| P24941 | 44 | Cyclin-dependent kinase 2 | CDK2 |
| P01137 | 42 | Transforming growth factor beta-1 proprotein | TGFB1 |
| P33993 | 41 | DNA replication licensing factor MCM7 | MCM7 |
| P35555 | 39 | Fibrillin-1 | FBN1 |
| P02771 | 39 | Alpha-fetoprotein | AFP |

**Table S4 A list of 400 up-regulated significantly differentially expressed proteins between the hypoxia-treatment group and the control group.**

| **Accession** | **Protein Name** | **Gene Name** | **Fold change** |
| --- | --- | --- | --- |
| Q9BY07 | Electrogenic sodium bicarbonate cotransporter 4 | SLC4A5 | 2.91 |
| Q5VUM1 | Succinate dehydrogenase assembly factor 4, mitochondrial | SDHAF4 | 2.65 |
| O00767 | Acyl-CoA desaturase | SCD | 2.13 |
| P08493 | Matrix Gla protein | MGP | 2.12 |
| P18859 | ATP synthase-coupling factor 6, mitochondrial | ATP5PF | 2.04 |
| Q5SZL2 | Centrosomal protein of 85 kDa-like | CEP85L | 2.02 |
| P15260 | Interferon gamma receptor 1 | IFNGR1 | 1.98 |
| Q9UPG8 | Zinc finger protein PLAGL2 | PLAGL2 | 1.96 |
| P59768 | Guanine nucleotide-binding protein G(I)/G(S)/G(O) subunit gamma-2 | GNG2 | 1.93 |
| P07602 | Prosaposin | PSAP | 1.86 |
| P80723 | Brain acid soluble protein 1 | BASP1 | 1.84 |
| Q13501 | Sequestosome-1 | SQSTM1 | 1.79 |
| Q4V9L6 | Transmembrane protein 119 | TMEM119 | 1.79 |
| Q9NRP0 | Oligosaccharyltransferase complex subunit OSTC | OSTC | 1.78 |
| P31949 | Protein S100-A11 | S100A11 | 1.78 |
| Q9BXB1 | Leucine-rich repeat-containing G-protein coupled receptor 4 | LGR4 | 1.77 |
| Q13772 | Nuclear receptor coactivator 4 | NCOA4 | 1.75 |
| Q13286 | Battenin | CLN3 | 1.72 |
| Q9P1F3 | Costars family protein ABRACL | ABRACL | 1.71 |
| Q96QD8 | Sodium-coupled neutral amino acid transporter 2 | SLC38A2 | 1.68 |
| P17936 | Insulin-like growth factor-binding protein 3 | IGFBP3 | 1.68 |
| Q9BT67 | NEDD4 family-interacting protein 1 | NDFIP1 | 1.67 |
| Q8TB45 | DEP domain-containing mTOR-interacting protein | DEPTOR | 1.66 |
| P14209 | CD99 antigen | CD99 | 1.66 |
| Q8TBF8 | Protein FAM81A | FAM81A | 1.65 |
| Q9UBI6 | Guanine nucleotide-binding protein G(I)/G(S)/G(O) subunit gamma-12 | GNG12 | 1.64 |
| Q8NFA0 | Ubiquitin carboxyl-terminal hydrolase 32 | USP32 | 1.64 |
| O60220 | Mitochondrial import inner membrane translocase subunit Tim8 A | TIMM8A | 1.63 |
| P10124 | Serglycin | SRGN | 1.63 |
| Q9NQX7 | Integral membrane protein 2C | ITM2C | 1.62 |
| Q7Z4F1 | Low-density lipoprotein receptor-related protein 10 | LRP10 | 1.61 |
| Q9P1Z2 | Calcium-binding and coiled-coil domain-containing protein 1 | CALCOCO1 | 1.61 |
| P60903 | Protein S100-A10 | S100A10 | 1.60 |
| Q9Y287 | Integral membrane protein 2B | ITM2B | 1.60 |
| O14817 | Tetraspanin-4 | TSPAN4 | 1.59 |
| A6NCE7 | Microtubule-associated proteins 1A/1B light chain 3 beta 2 | MAP1LC3B2 | 1.59 |
| Q8N4H5 | Mitochondrial import receptor subunit TOM5 homolog | TOMM5 | 1.59 |
| P02792 | Ferritin light chain | FTL | 1.55 |
| O14561 | Acyl carrier protein, mitochondrial | NDUFAB1 | 1.54 |
| P35442 | Thrombospondin-2 | THBS2 | 1.54 |
| Q8TCY9 | Up-regulator of cell proliferation | URGCP | 1.53 |
| Q53EL6 | Programmed cell death protein 4 | PDCD4 | 1.51 |
| P20962 | Parathymosin | PTMS | 1.51 |
| Q8TCZ2 | CD99 antigen-like protein 2 | CD99L2 | 1.51 |
| Q7Z7L8 | Uncharacterized protein C11orf96 | C11orf96 | 1.50 |
| P56277 | Cx9C motif-containing protein 4 | CMC4 | 1.50 |
| Q9Y5J9 | Mitochondrial import inner membrane translocase subunit Tim8 B | TIMM8B | 1.50 |
| Q13530 | Serine incorporator 3 | SERINC3 | 1.49 |
| A0A0U1RRE5 | Negative regulator of P-body association | NBDY | 1.48 |
| Q68BL8 | Olfactomedin-like protein 2B | OLFML2B | 1.48 |
| P05386 | 60S acidic ribosomal protein P1 | RPLP1 | 1.47 |
| Q13541 | Eukaryotic translation initiation factor 4E-binding protein 1 | EIF4EBP1 | 1.47 |
| Q15532 | Protein SSXT | SS18 | 1.47 |
| Q86VP1 | Tax1-binding protein 1 | TAX1BP1 | 1.47 |
| P17677 | Neuromodulin | GAP43 | 1.46 |
| O43768 | Alpha-endosulfine | ENSA | 1.46 |
| P52815 | 39S ribosomal protein L12, mitochondrial | MRPL12 | 1.46 |
| P10620 | Microsomal glutathione S-transferase 1 | MGST1 | 1.46 |
| P10599 | Thioredoxin | TXN | 1.45 |
| P07585 | Decorin | DCN | 1.45 |
| Q15012 | Lysosomal-associated transmembrane protein 4A | LAPTM4A | 1.44 |
| Q5JTB6 | Placenta-specific protein 9 | PLAC9 | 1.44 |
| P15954 | Cytochrome c oxidase subunit 7C, mitochondrial | COX7C | 1.44 |
| Q13938 | Calcyphosin | CAPS | 1.44 |
| P03928 | ATP synthase protein 8 | MT-ATP8 | 1.43 |
| P17302 | Gap junction alpha-1 protein | GJA1 | 1.43 |
| P13611 | Versican core protein | VCAN | 1.43 |
| P07919 | Cytochrome b-c1 complex subunit 6, mitochondrial | UQCRH | 1.43 |
| P16234 | Platelet-derived growth factor receptor alpha | PDGFRA | 1.43 |
| Q4ZHG4 | Fibronectin type III domain-containing protein 1 | FNDC1 | 1.43 |
| Q08629 | Testican-1 | SPOCK1 | 1.42 |
| Q93077 | Histone H2A type 1-C | HIST1H2AC | 1.42 |
| O95477 | Phospholipid-transporting ATPase ABCA1 | ABCA1 | 1.41 |
| P60709 | Actin, cytoplasmic 1 | ACTB | 1.41 |
| P63098 | Calcineurin subunit B type 1 | PPP3R1 | 1.41 |
| P05387 | 60S acidic ribosomal protein P2 | RPLP2 | 1.41 |
| P16035 | Metalloproteinase inhibitor 2 | TIMP2 | 1.41 |
| Q8N111 | Cell cycle exit and neuronal differentiation protein 1 | CEND1 | 1.41 |
| Q14596 | Next to BRCA1 gene 1 protein | NBR1 | 1.41 |
| P03891 | NADH-ubiquinone oxidoreductase chain 2 | MT-ND2 | 1.41 |
| P84157 | Matrix-remodeling-associated protein 7 | MXRA7 | 1.41 |
| Q5T5N4 | Uncharacterized protein C6orf118 | C6orf118 | 1.40 |
| P42773 | Cyclin-dependent kinase 4 inhibitor C | CDKN2C | 1.40 |
| P37173 | TGF-beta receptor type-2 | TGFBR2 | 1.40 |
| Q8WWI5 | Choline transporter-like protein 1 | SLC44A1 | 1.40 |
| Q8WUW1 | Protein BRICK1 | BRK1 | 1.40 |
| Q9UK76 | Jupiter microtubule associated homolog 1 | JPT1 | 1.40 |
| P07108 | Acyl-CoA-binding protein | DBI | 1.40 |
| Q06481 | Amyloid-like protein 2 | APLP2 | 1.40 |
| P09497 | Clathrin light chain B | CLTB | 1.39 |
| Q9BXS4 | Transmembrane protein 59 | TMEM59 | 1.39 |
| P19021 | Peptidyl-glycine alpha-amidating monooxygenase | PAM | 1.39 |
| Q16832 | Discoidin domain-containing receptor 2 | DDR2 | 1.39 |
| P09619 | Platelet-derived growth factor receptor beta | PDGFRB | 1.39 |
| Q9NZA1 | Chloride intracellular channel protein 5 | CLIC5 | 1.38 |
| P01210 | Proenkephalin-A | PENK | 1.38 |
| P15529 | Membrane cofactor protein | CD46 | 1.38 |
| P63261 | Actin, cytoplasmic 2 | ACTG1 | 1.38 |
| Q5T655 | Cilia- and flagella-associated protein 58 | CFAP58 | 1.38 |
| Q5VZF2 | Muscleblind-like protein 2 | MBNL2 | 1.38 |
| P02462 | Collagen alpha-1(IV) chain | COL4A1 | 1.38 |
| P22676 | Calretinin | CALB2 | 1.37 |
| Q9UKB1 | F-box/WD repeat-containing protein 11 | FBXW11 | 1.37 |
| P05413 | Fatty acid-binding protein, heart | FABP3 | 1.37 |
| Q9UBN6 | Tumor necrosis factor receptor superfamily member 10D | TNFRSF10D | 1.37 |
| O00115 | Deoxyribonuclease-2-alpha | DNASE2 | 1.37 |
| P35754 | Glutaredoxin-1 | GLRX | 1.37 |
| Q9BTT4 | Mediator of RNA polymerase II transcription subunit 10 | MED10 | 1.37 |
| Q99576 | TSC22 domain family protein 3 | TSC22D3 | 1.37 |
| C9JRZ8 | Aldo-keto reductase family 1 member B15 | AKR1B15 | 1.37 |
| P56817 | Beta-secretase 1 | BACE1 | 1.36 |
| Q14393 | Growth arrest-specific protein 6 | GAS6 | 1.36 |
| Q8NC42 | E3 ubiquitin-protein ligase RNF149 | RNF149 | 1.36 |
| P29373 | Cellular retinoic acid-binding protein 2 | CRABP2 | 1.36 |
| P13987 | CD59 glycoprotein | CD59 | 1.36 |
| Q9Y4K0 | Lysyl oxidase homolog 2 | LOXL2 | 1.36 |
| Q9NUN5 | Probable lysosomal cobalamin transporter | LMBRD1 | 1.36 |
| Q9NVA4 | Transmembrane protein 184C | TMEM184C | 1.36 |
| Q9BRK3 | Matrix remodeling-associated protein 8 | MXRA8 | 1.35 |
| Q8TAD7 | Overexpressed in colon carcinoma 1 protein | OCC1 | 1.35 |
| O14763 | Tumor necrosis factor receptor superfamily member 10B | TNFRSF10B | 1.35 |
| Q9HAB3 | Solute carrier family 52, riboflavin transporter, member 2 | SLC52A2 | 1.35 |
| P09496 | Clathrin light chain A | CLTA | 1.35 |
| P08582 | Melanotransferrin | MELTF | 1.35 |
| Q9NPF0 | CD320 antigen | CD320 | 1.35 |
| Q92597 | Protein NDRG1 | NDRG1 | 1.35 |
| Q08334 | Interleukin-10 receptor subunit beta | IL10RB | 1.34 |
| O43399 | Tumor protein D54 | TPD52L2 | 1.34 |
| P08195 | 4F2 cell-surface antigen heavy chain | SLC3A2 | 1.34 |
| P00736 | Complement C1r subcomponent | C1R | 1.34 |
| Q99471 | Prefoldin subunit 5 | PFDN5 | 1.34 |
| Q8NC54 | Keratinocyte-associated transmembrane protein 2 | KCT2 | 1.34 |
| O95965 | Integrin beta-like protein 1 | ITGBL1 | 1.34 |
| Q6UXH9 | Inactive serine protease PAMR1 | PAMR1 | 1.34 |
| P08473 | Neprilysin | MME | 1.34 |
| P08253 | 72 kDa type IV collagenase | MMP2 | 1.34 |
| P28799 | Progranulin | GRN | 1.34 |
| P05204 | Non-histone chromosomal protein HMG-17 | HMGN2 | 1.34 |
| Q8IZJ1 | Netrin receptor UNC5B | UNC5B | 1.34 |
| Q9HC62 | Sentrin-specific protease 2 | SENP2 | 1.34 |
| O75629 | Protein CREG1 | CREG1 | 1.34 |
| Q9BXV9 | EKC/KEOPS complex subunit GON7 | GON7 | 1.34 |
| P11279 | Lysosome-associated membrane glycoprotein 1 | LAMP1 | 1.34 |
| P08651 | Nuclear factor 1 C-type | NFIC | 1.34 |
| Q7Z3Y7 | Keratin, type I cytoskeletal 28 | KRT28 | 1.33 |
| O43677 | NADH dehydrogenase [ubiquinone] 1 subunit C1, mitochondrial | NDUFC1 | 1.33 |
| Q9NQX5 | Neural proliferation differentiation and control protein 1 | NPDC1 | 1.33 |
| Q8TBP5 | Membrane protein FAM174A | FAM174A | 1.33 |
| P10909 | Clusterin | CLU | 1.33 |
| Q9NYC9 | Dynein heavy chain 9, axonemal | DNAH9 | 1.33 |
| O95182 | NADH dehydrogenase [ubiquinone] 1 alpha subcomplex subunit 7 | NDUFA7 | 1.33 |
| Q6ZSJ8 | Uncharacterized protein C1orf122 | C1orf122 | 1.33 |
| P62873 | Guanine nucleotide-binding protein G(I)/G(S)/G(T) subunit beta-1 | GNB1 | 1.33 |
| Q86UU0 | B-cell CLL/lymphoma 9-like protein | BCL9L | 1.33 |
| Q969G5 | Caveolae-associated protein 3 | CAVIN3 | 1.33 |
| Q8NFJ5 | Retinoic acid-induced protein 3 | GPRC5A | 1.32 |
| P55287 | Cadherin-11 | CDH11 | 1.32 |
| P02794 | Ferritin heavy chain | FTH1 | 1.32 |
| Q93096 | Protein tyrosine phosphatase type IVA 1 | PTP4A1 | 1.32 |
| P36954 | DNA-directed RNA polymerase II subunit RPB9 | POLR2I | 1.32 |
| Q969E4 | Transcription elongation factor A protein-like 3 | TCEAL3 | 1.32 |
| Q9H0X4 | Protein FAM234A | FAM234A | 1.32 |
| Q9HCL0 | Protocadherin-18 | PCDH18 | 1.32 |
| P10114 | Ras-related protein Rap-2a | RAP2A | 1.32 |
| Q9BXJ0 | Complement C1q tumor necrosis factor-related protein 5 | C1QTNF5 | 1.32 |
| P62879 | Guanine nucleotide-binding protein G(I)/G(S)/G(T) subunit beta-2 | GNB2 | 1.32 |
| Q9Y240 | C-type lectin domain family 11 member A | CLEC11A | 1.32 |
| O60330 | Protocadherin gamma-A12 | PCDHGA12 | 1.32 |
| O00560 | Syntenin-1 | SDCBP | 1.32 |
| Q96B23 | Uncharacterized protein C18orf25 | C18orf25 | 1.32 |
| P20674 | Cytochrome c oxidase subunit 5A, mitochondrial | COX5A | 1.32 |
| Q05639 | Elongation factor 1-alpha 2 | EEF1A2 | 1.32 |
| Q9Y5J5 | Pleckstrin homology-like domain family A member 3 | PHLDA3 | 1.32 |
| Q9Y3E1 | Hepatoma-derived growth factor-related protein 3 | HDGFL3 | 1.32 |
| Q9Y478 | 5'-AMP-activated protein kinase subunit beta-1 | PRKAB1 | 1.32 |
| O14495 | Phospholipid phosphatase 3 | PLPP3 | 1.32 |
| P16070 | CD44 antigen | CD44 | 1.31 |
| P04921 | Glycophorin-C | GYPC | 1.31 |
| P56211 | cAMP-regulated phosphoprotein 19 | ARPP19 | 1.31 |
| P62328 | Thymosin beta-4 | TMSB4X | 1.31 |
| P05114 | Non-histone chromosomal protein HMG-14 | HMGN1 | 1.31 |
| Q9ULI3 | Protein HEG homolog 1 | HEG1 | 1.31 |
| Q15847 | Adipogenesis regulatory factor | ADIRF | 1.31 |
| P84090 | Enhancer of rudimentary homolog | ERH | 1.31 |
| P63218 | Guanine nucleotide-binding protein G(I)/G(S)/G(O) subunit gamma-5 | GNG5 | 1.31 |
| Q16626 | Male-enhanced antigen 1 | MEA1 | 1.31 |
| Q6PKC3 | Thioredoxin domain-containing protein 11 | TXNDC11 | 1.31 |
| P37235 | Hippocalcin-like protein 1 | HPCAL1 | 1.30 |
| Q9BQJ4 | Transmembrane protein 47 | TMEM47 | 1.30 |
| P50281 | Matrix metalloproteinase-14 | MMP14 | 1.30 |
| Q92743 | Serine protease HTRA1 | HTRA1 | 1.30 |
| Q05707 | Collagen alpha-1(XIV) chain | COL14A1 | 1.30 |
| Q14257 | Reticulocalbin-2 | RCN2 | 1.30 |
| Q969T3 | Sorting nexin-21 | SNX21 | 1.30 |
| P34925 | Tyrosine-protein kinase RYK | RYK | 1.30 |
| P06454 | Prothymosin alpha | PTMA | 1.30 |
| Q8IXM2 | Chromatin complexes subunit BAP18 | BAP18 | 1.30 |
| P40189 | Interleukin-6 receptor subunit beta | IL6ST | 1.30 |
| O60888 | Protein CutA | CUTA | 1.30 |
| P42702 | Leukemia inhibitory factor receptor | LIFR | 1.30 |
| Q9UPX6 | Major intrinsically disordered Notch2-binding receptor 1 | MINAR1 | 1.30 |
| P29966 | Myristoylated alanine-rich C-kinase substrate | MARCKS | 1.29 |
| Q9UBT6 | DNA polymerase kappa | POLK | 1.29 |
| Q5ZPR3 | CD276 antigen | CD276 | 1.29 |
| O95183 | Vesicle-associated membrane protein 5 | VAMP5 | 1.29 |
| Q13393 | Phospholipase D1 | PLD1 | 1.29 |
| Q9NYJ1 | Cytochrome c oxidase assembly factor 4 homolog, mitochondrial | COA4 | 1.29 |
| Q8N3F0 | Maturin | MTURN | 1.29 |
| P09871 | Complement C1s subcomponent | C1S | 1.29 |
| P06703 | Protein S100-A6 | S100A6 | 1.29 |
| Q86VE9 | Serine incorporator 5 | SERINC5 | 1.29 |
| Q6ZSZ5 | Rho guanine nucleotide exchange factor 18 | ARHGEF18 | 1.29 |
| P0CG48 | Polyubiquitin-C | UBC | 1.29 |
| P30626 | Sorcin | SRI | 1.29 |
| Q9P2D0 | Inhibitor of Bruton tyrosine kinase | IBTK | 1.29 |
| Q14129 | Protein DGCR6 | DGCR6 | 1.28 |
| Q6QNY1 | Biogenesis of lysosome-related organelles complex 1 subunit 2 | BLOC1S2 | 1.28 |
| Q10588 | ADP-ribosyl cyclase/cyclic ADP-ribose hydrolase 2 | BST1 | 1.28 |
| Q9UBV8 | Peflin | PEF1 | 1.28 |
| Q9H773 | dCTP pyrophosphatase 1 | DCTPP1 | 1.28 |
| Q9H8H3 | Methyltransferase-like protein 7A | METTL7A | 1.28 |
| O75947 | ATP synthase subunit d, mitochondrial | ATP5PD | 1.28 |
| Q9C0H2 | Protein tweety homolog 3 | TTYH3 | 1.28 |
| P55145 | Mesencephalic astrocyte-derived neurotrophic factor | MANF | 1.28 |
| P20742 | Pregnancy zone protein | PZP | 1.28 |
| Q4KWH8 | 1-phosphatidylinositol 4,5-bisphosphate phosphodiesterase eta-1 | PLCH1 | 1.28 |
| A5YM69 | Rho guanine nucleotide exchange factor 35 | ARHGEF35 | 1.28 |
| P54289 | Voltage-dependent calcium channel subunit alpha-2/delta-1 | CACNA2D1 | 1.28 |
| Q9Y275 | Tumor necrosis factor ligand superfamily member 13B | TNFSF13B | 1.28 |
| P53801 | Pituitary tumor-transforming gene 1 protein-interacting protein | PTTG1IP | 1.28 |
| P60602 | Reactive oxygen species modulator 1 | ROMO1 | 1.27 |
| P10176 | Cytochrome c oxidase subunit 8A, mitochondrial | COX8A | 1.27 |
| O15212 | Prefoldin subunit 6 | PFDN6 | 1.27 |
| Q8NHU3 | Phosphatidylcholine:ceramide cholinephosphotransferase 2 | SGMS2 | 1.27 |
| Q16718 | NADH dehydrogenase [ubiquinone] 1 alpha subcomplex subunit 5 | NDUFA5 | 1.27 |
| P11166 | Solute carrier family 2, facilitated glucose transporter member 1 | SLC2A1 | 1.27 |
| Q16678 | Cytochrome P450 1B1 | CYP1B1 | 1.27 |
| Q9BXY0 | Protein MAK16 homolog | MAK16 | 1.27 |
| P27487 | Dipeptidyl peptidase 4 | DPP4 | 1.27 |
| Q9H6X2 | Anthrax toxin receptor 1 | ANTXR1 | 1.27 |
| P07237 | Protein disulfide-isomerase | P4HB | 1.27 |
| Q13137 | Calcium-binding and coiled-coil domain-containing protein 2 | CALCOCO2 | 1.27 |
| P34741 | Syndecan-2 | SDC2 | 1.27 |
| P48509 | CD151 antigen | CD151 | 1.27 |
| Q969Z3 | Mitochondrial amidoxime reducing component 2 | MARC2 | 1.27 |
| P27797 | Calreticulin | CALR | 1.26 |
| Q9UMX5 | Neudesin | NENF | 1.26 |
| P35869 | Aryl hydrocarbon receptor | AHR | 1.26 |
| Q08431 | Lactadherin | MFGE8 | 1.26 |
| Q14956 | Transmembrane glycoprotein NMB | GPNMB | 1.26 |
| Q9NQP4 | Prefoldin subunit 4 | PFDN4 | 1.26 |
| Q16890 | Tumor protein D53 | TPD52L1 | 1.26 |
| O43567 | E3 ubiquitin-protein ligase RNF13 | RNF13 | 1.26 |
| P49006 | MARCKS-related protein | MARCKSL1 | 1.26 |
| P17931 | Galectin-3 | LGALS3 | 1.26 |
| Q9BQD3 | KxDL motif-containing protein 1 | KXD1 | 1.26 |
| Q9NRX5 | Serine incorporator 1 | SERINC1 | 1.26 |
| Q9BPZ3 | Polyadenylate-binding protein-interacting protein 2 | PAIP2 | 1.26 |
| Q6UY14 | ADAMTS-like protein 4 | ADAMTSL4 | 1.26 |
| P33240 | Cleavage stimulation factor subunit 2 | CSTF2 | 1.26 |
| Q14534 | Squalene monooxygenase | SQLE | 1.26 |
| P19256 | Lymphocyte function-associated antigen 3 | CD58 | 1.26 |
| Q96D15 | Reticulocalbin-3 | RCN3 | 1.26 |
| P41208 | Centrin-2 | CETN2 | 1.25 |
| O00479 | High mobility group nucleosome-binding domain-containing protein 4 | HMGN4 | 1.25 |
| Q8NFD5 | AT-rich interactive domain-containing protein 1B | ARID1B | 1.25 |
| O75094 | Slit homolog 3 protein | SLIT3 | 1.25 |
| P58546 | Myotrophin | MTPN | 1.25 |
| P51858 | Hepatoma-derived growth factor | HDGF | 1.25 |
| P30044 | Peroxiredoxin-5, mitochondrial | PRDX5 | 1.25 |
| P55290 | Cadherin-13 | CDH13 | 1.25 |
| Q9NRR5 | Ubiquilin-4 | UBQLN4 | 1.25 |
| Q9Y3C5 | RING finger protein 11 | RNF11 | 1.25 |
| Q9Y5U9 | Immediate early response 3-interacting protein 1 | IER3IP1 | 1.25 |
| Q7Z7N9 | Transmembrane protein 179B | TMEM179B | 1.25 |
| O60637 | Tetraspanin-3 | TSPAN3 | 1.25 |
| O15050 | TPR and ankyrin repeat-containing protein 1 | TRANK1 | 1.25 |
| Q9Y5J7 | Mitochondrial import inner membrane translocase subunit Tim9 | TIMM9 | 1.25 |
| P04439 | HLA class I histocompatibility antigen, A alpha chain | HLA-A | 1.25 |
| Q9Y2R0 | Cytochrome c oxidase assembly factor 3 homolog, mitochondrial | COA3 | 1.25 |
| O95864 | Acyl-CoA 6-desaturase | FADS2 | 1.25 |
| Q8N129 | Protein canopy homolog 4 | CNPY4 | 1.24 |
| O95562 | Vesicle transport protein SFT2B | SFT2D2 | 1.24 |
| Q9Y639 | Neuroplastin | NPTN | 1.24 |
| P55058 | Phospholipid transfer protein | PLTP | 1.24 |
| Q96AQ6 | Pre-B-cell leukemia transcription factor-interacting protein 1 | PBXIP1 | 1.24 |
| O00264 | Membrane-associated progesterone receptor component 1 | PGRMC1 | 1.24 |
| P04179 | Superoxide dismutase [Mn], mitochondrial | SOD2 | 1.24 |
| P08962 | CD63 antigen | CD63 | 1.24 |
| Q6UXH1 | Protein disulfide isomerase CRELD2 | CRELD2 | 1.24 |
| O00478 | Butyrophilin subfamily 3 member A3 | BTN3A3 | 1.24 |
| Q9H497 | Torsin-3A | TOR3A | 1.24 |
| Q9BX68 | Histidine triad nucleotide-binding protein 2, mitochondrial | HINT2 | 1.24 |
| P13497 | Bone morphogenetic protein 1 | BMP1 | 1.24 |
| P20810 | Calpastatin | CAST | 1.24 |
| P27105 | Erythrocyte band 7 integral membrane protein | STOM | 1.24 |
| P35613 | Basigin | BSG | 1.24 |
| P60174 | Triosephosphate isomerase | TPI1 | 1.24 |
| O60238 | BCL2/adenovirus E1B 19 kDa protein-interacting protein 3-like | BNIP3L | 1.24 |
| O14641 | Segment polarity protein dishevelled homolog DVL-2 | DVL2 | 1.24 |
| O75072 | Fukutin | FKTN | 1.24 |
| Q9Y4Y9 | U6 snRNA-associated Sm-like protein LSm5 | LSM5 | 1.24 |
| P14174 | Macrophage migration inhibitory factor | MIF | 1.24 |
| Q06136 | 3-ketodihydrosphingosine reductase | KDSR | 1.24 |
| Q13336 | Urea transporter 1 | SLC14A1 | 1.24 |
| Q2TAM9 | Tumor suppressor candidate gene 1 protein | TUSC1 | 1.23 |
| P08842 | Steryl-sulfatase | STS | 1.23 |
| P46091 | G-protein coupled receptor 1 | GPR1 | 1.23 |
| Q5VW38 | Protein GPR107 | GPR107 | 1.23 |
| Q13445 | Transmembrane emp24 domain-containing protein 1 | TMED1 | 1.23 |
| Q9NRQ2 | Phospholipid scramblase 4 | PLSCR4 | 1.23 |
| P23434 | Glycine cleavage system H protein, mitochondrial | GCSH | 1.23 |
| Q96CM8 | Medium-chain acyl-CoA ligase ACSF2, mitochondrial | ACSF2 | 1.23 |
| Q7Z422 | SUZ domain-containing protein 1 | SZRD1 | 1.23 |
| Q8NBR6 | Ubiquitin carboxyl-terminal hydrolase MINDY-2 | MINDY2 | 1.23 |
| P09603 | Macrophage colony-stimulating factor 1 | CSF1 | 1.23 |
| Q13332 | Receptor-type tyrosine-protein phosphatase S | PTPRS | 1.23 |
| Q8N5C1 | Calcium homeostasis modulator protein 5 | CALHM5 | 1.23 |
| P05067 | Amyloid-beta precursor protein | APP | 1.23 |
| L0R6Q1 | SLC35A4 upstream open reading frame protein | SLC35A4 | 1.23 |
| Q15063 | Periostin | POSTN | 1.23 |
| P61601 | Neurocalcin-delta | NCALD | 1.23 |
| Q9UI08 | Ena/VASP-like protein | EVL | 1.23 |
| Q8WTV0 | Scavenger receptor class B member 1 | SCARB1 | 1.23 |
| P25963 | NF-kappa-B inhibitor alpha | NFKBIA | 1.23 |
| P13473 | Lysosome-associated membrane glycoprotein 2 | LAMP2 | 1.23 |
| Q99584 | Protein S100-A13 | S100A13 | 1.23 |
| O60613 | Selenoprotein F | SELENOF | 1.23 |
| O43716 | Glutamyl-tRNA(Gln) amidotransferase subunit C, mitochondrial | GATC | 1.23 |
| P17813 | Endoglin | ENG | 1.23 |
| P0C7P3 | Protein SLFN14 | SLFN14 | 1.23 |
| O75438 | NADH dehydrogenase [ubiquinone] 1 beta subcomplex subunit 1 | NDUFB1 | 1.22 |
| Q9Y6Q2 | Stonin-1 | STON1 | 1.22 |
| Q92542 | Nicastrin | NCSTN | 1.22 |
| Q9UKY7 | Protein CDV3 homolog | CDV3 | 1.22 |
| Q9UFG5 | UPF0449 protein C19orf25 | C19orf25 | 1.22 |
| P04216 | Thy-1 membrane glycoprotein | THY1 | 1.22 |
| P27658 | Collagen alpha-1(VIII) chain | COL8A1 | 1.22 |
| O15031 | Plexin-B2 | PLXNB2 | 1.22 |
| Q9BXT2 | Voltage-dependent calcium channel gamma-6 subunit | CACNG6 | 1.22 |
| Q9P2B2 | Prostaglandin F2 receptor negative regulator | PTGFRN | 1.22 |
| Q9UHV9 | Prefoldin subunit 2 | PFDN2 | 1.22 |
| O95302 | Peptidyl-prolyl cis-trans isomerase FKBP9 | FKBP9 | 1.22 |
| Q86YS7 | C2 domain-containing protein 5 | C2CD5 | 1.22 |
| P42771 | Cyclin-dependent kinase inhibitor 2A | CDKN2A | 1.22 |
| P17275 | Transcription factor jun-B | JUNB | 1.22 |
| Q9P1T7 | MyoD family inhibitor domain-containing protein | MDFIC | 1.22 |
| Q7L1V2 | Vacuolar fusion protein MON1 homolog B | MON1B | 1.22 |
| Q9Y5Z0 | Beta-secretase 2 | BACE2 | 1.22 |
| P08581 | Hepatocyte growth factor receptor | MET | 1.22 |
| Q9ULG6 | Cell cycle progression protein 1 | CCPG1 | 1.22 |
| P35555 | Fibrillin-1 | FBN1 | 1.22 |
| Q92551 | Inositol hexakisphosphate kinase 1 | IP6K1 | 1.22 |
| Q8TCT8 | Signal peptide peptidase-like 2A | SPPL2A | 1.22 |
| P06756 | Integrin alpha-V | ITGAV | 1.22 |
| Q92629 | Delta-sarcoglycan | SGCD | 1.22 |
| O95297 | Myelin protein zero-like protein 1 | MPZL1 | 1.22 |
| Q06323 | Proteasome activator complex subunit 1 | PSME1 | 1.22 |
| P60660 | Myosin light polypeptide 6 | MYL6 | 1.22 |
| Q96EQ0 | Small glutamine-rich tetratricopeptide repeat-containing protein beta | SGTB | 1.21 |
| P04920 | Anion exchange protein 2 | SLC4A2 | 1.21 |
| O15118 | NPC intracellular cholesterol transporter 1 | NPC1 | 1.21 |
| P42126 | Enoyl-CoA delta isomerase 1, mitochondrial | ECI1 | 1.21 |
| Q9UPN9 | E3 ubiquitin-protein ligase TRIM33 | TRIM33 | 1.21 |
| P14406 | Cytochrome c oxidase subunit 7A2, mitochondrial | COX7A2 | 1.21 |
| Q9Y5Z4 | Heme-binding protein 2 | HEBP2 | 1.21 |
| P54727 | UV excision repair protein RAD23 homolog B | RAD23B | 1.21 |
| P31512 | Dimethylaniline monooxygenase [N-oxide-forming] 4 | FMO4 | 1.21 |
| P53384 | Cytosolic Fe-S cluster assembly factor NUBP1 | NUBP1 | 1.21 |
| Q9UKU9 | Angiopoietin-related protein 2 | ANGPTL2 | 1.21 |
| Q12884 | Prolyl endopeptidase FAP | FAP | 1.21 |
| O15394 | Neural cell adhesion molecule 2 | NCAM2 | 1.21 |
| P02751 | Fibronectin | FN1 | 1.21 |
| Q13740 | CD166 antigen | ALCAM | 1.21 |
| P14314 | Glucosidase 2 subunit beta | PRKCSH | 1.21 |
| Q99653 | Calcineurin B homologous protein 1 | CHP1 | 1.21 |
| P27701 | CD82 antigen | CD82 | 1.21 |
| P61956 | Small ubiquitin-related modifier 2 | SUMO2 | 1.21 |
| Q7Z398 | Zinc finger protein 550 | ZNF550 | 1.21 |
| O15511 | Actin-related protein 2/3 complex subunit 5 | ARPC5 | 1.21 |
| Q96IK1 | Biorientation of chromosomes in cell division protein 1 | BOD1 | 1.21 |
| O15173 | Membrane-associated progesterone receptor component 2 | PGRMC2 | 1.21 |
| O75874 | Isocitrate dehydrogenase [NADP] cytoplasmic | IDH1 | 1.21 |
| Q9BRT2 | Ubiquinol-cytochrome-c reductase complex assembly factor 2 | UQCC2 | 1.21 |
| Q92688 | Acidic leucine-rich nuclear phosphoprotein 32 family member B | ANP32B | 1.21 |
| P60520 | Gamma-aminobutyric acid receptor-associated protein-like 2 | GABARAPL2 | 1.20 |
| Q9BWD1 | Acetyl-CoA acetyltransferase, cytosolic | ACAT2 | 1.20 |
| Q9ULI0 | ATPase family AAA domain-containing protein 2B | ATAD2B | 1.20 |
| P24310 | Cytochrome c oxidase subunit 7A1, mitochondrial | COX7A1 | 1.20 |
| Q9NX08 | COMM domain-containing protein 8 | COMMD8 | 1.20 |
| Q96FQ6 | Protein S100-A16 | S100A16 | 1.20 |
| P24821 | Tenascin | TNC | 1.20 |
| P63313 | Thymosin beta-10 | TMSB10 | 1.20 |
| P27449 | V-type proton ATPase 16 kDa proteolipid subunit | ATP6V0C | 1.20 |
| Q07092 | Collagen alpha-1(XVI) chain | COL16A1 | 1.20 |
| O60462 | Neuropilin-2 | NRP2 | 1.20 |
| Q9HBR0 | Putative sodium-coupled neutral amino acid transporter 10 | SLC38A10 | 1.20 |
| Q9NZV1 | Cysteine-rich motor neuron 1 protein | CRIM1 | 1.20 |
| P01137 | Transforming growth factor beta-1 proprotein | TGFB1 | 1.20 |
| Q96AY3 | Peptidyl-prolyl cis-trans isomerase FKBP10 | FKBP10 | 1.20 |

**Table S5 A list of 286 down-regulated significantly differentially expressed proteins between the hypoxia-treatment group and the control group.**

| **Accession** | **Protein Name** | **Gene Name** | **Fold change** |
| --- | --- | --- | --- |
| P0DP58 | Ly-6/neurotoxin-like protein 1 | LYNX1 | 0.83 |
| Q9ULT8 | E3 ubiquitin-protein ligase HECTD1 | HECTD1 | 0.83 |
| O95793 | Double-stranded RNA-binding protein Staufen homolog 1 | STAU1 | 0.83 |
| Q9UHE8 | Metalloreductase STEAP1 | STEAP1 | 0.83 |
| Q9BZD4 | Kinetochore protein Nuf2 | NUF2 | 0.83 |
| O75494 | Serine/arginine-rich splicing factor 10 | SRSF10 | 0.83 |
| Q14644 | Ras GTPase-activating protein 3 | RASA3 | 0.83 |
| Q9H2D1 | Mitochondrial folate transporter/carrier | SLC25A32 | 0.83 |
| Q99707 | Methionine synthase | MTR | 0.83 |
| O95801 | Tetratricopeptide repeat protein 4 | TTC4 | 0.83 |
| Q8IVB5 | LIX1-like protein | LIX1L | 0.83 |
| O15121 | Sphingolipid delta(4)-desaturase DES1 | DEGS1 | 0.83 |
| Q7Z417 | Nuclear fragile X mental retardation-interacting protein 2 | NUFIP2 | 0.83 |
| P48730 | Casein kinase I isoform delta | CSNK1D | 0.83 |
| P42338 | Phosphatidylinositol 4,5-bisphosphate 3-kinase catalytic subunit beta isoform | PIK3CB | 0.83 |
| Q9NPB0 | SAYSvFN domain-containing protein 1 | SAYSD1 | 0.83 |
| Q9P031 | Thyroid transcription factor 1-associated protein 26 | CCDC59 | 0.83 |
| Q9BWL3 | Uncharacterized protein C1orf43 | C1orf43 | 0.83 |
| Q8WV24 | Pleckstrin homology-like domain family A member 1 | PHLDA1 | 0.83 |
| P18858 | DNA ligase 1 | LIG1 | 0.83 |
| Q9UHR6 | Zinc finger HIT domain-containing protein 2 | ZNHIT2 | 0.83 |
| P33993 | DNA replication licensing factor MCM7 | MCM7 | 0.83 |
| Q9HAN9 | Nicotinamide/nicotinic acid mononucleotide adenylyltransferase 1 | NMNAT1 | 0.83 |
| P49023 | Paxillin | PXN | 0.83 |
| Q5TC84 | Opioid growth factor receptor-like protein 1 | OGFRL1 | 0.83 |
| Q6XZF7 | Dynamin-binding protein | DNMBP | 0.83 |
| P50897 | Palmitoyl-protein thioesterase 1 | PPT1 | 0.83 |
| Q9NUW8 | Tyrosyl-DNA phosphodiesterase 1 | TDP1 | 0.83 |
| Q14123 | Calcium/calmodulin-dependent 3',5'-cyclic nucleotide phosphodiesterase 1C | PDE1C | 0.83 |
| Q9Y232 | Chromodomain Y-like protein | CDYL | 0.83 |
| Q9BZX2 | Uridine-cytidine kinase 2 | UCK2 | 0.83 |
| P11387 | DNA topoisomerase 1 | TOP1 | 0.83 |
| Q9UJD0 | Regulating synaptic membrane exocytosis protein 3 | RIMS3 | 0.83 |
| Q9H501 | ESF1 homolog | ESF1 | 0.83 |
| Q86U86 | Protein polybromo-1 | PBRM1 | 0.83 |
| P49736 | DNA replication licensing factor MCM2 | MCM2 | 0.83 |
| Q9GZS1 | DNA-directed RNA polymerase I subunit RPA49 | POLR1E | 0.83 |
| Q6PD74 | Alpha- and gamma-adaptin-binding protein p34 | AAGAB | 0.83 |
| P58215 | Lysyl oxidase homolog 3 | LOXL3 | 0.83 |
| Q8IXK0 | Polyhomeotic-like protein 2 | PHC2 | 0.83 |
| O14646 | Chromodomain-helicase-DNA-binding protein 1 | CHD1 | 0.83 |
| Q6ZNA5 | Ferric-chelate reductase 1 | FRRS1 | 0.83 |
| Q92871 | Phosphomannomutase 1 | PMM1 | 0.82 |
| Q9H7V2 | Synapse differentiation-inducing gene protein 1 | SYNDIG1 | 0.82 |
| Q16637 | Survival motor neuron protein | SMN1 | 0.82 |
| P49767 | Vascular endothelial growth factor C | VEGFC | 0.82 |
| P29084 | Transcription initiation factor IIE subunit beta | GTF2E2 | 0.82 |
| P02675 | Fibrinogen beta chain | FGB | 0.82 |
| Q6P4A7 | Sideroflexin-4 | SFXN4 | 0.82 |
| P78316 | Nucleolar protein 14 | NOP14 | 0.82 |
| Q9GZZ1 | N-alpha-acetyltransferase 50 | NAA50 | 0.82 |
| Q8NC60 | Nitric oxide-associated protein 1 | NOA1 | 0.82 |
| Q8TCG1 | Protein CIP2A | CIP2A | 0.82 |
| Q9H8U3 | AN1-type zinc finger protein 3 | ZFAND3 | 0.82 |
| Q96HA8 | Protein N-terminal glutamine amidohydrolase | WDYHV1 | 0.82 |
| Q15398 | Disks large-associated protein 5 | DLGAP5 | 0.82 |
| Q13614 | Myotubularin-related protein 2 | MTMR2 | 0.82 |
| Q9Y3A2 | Probable U3 small nucleolar RNA-associated protein 11 | UTP11 | 0.82 |
| O00488 | Zinc finger protein 593 | ZNF593 | 0.82 |
| Q70JA7 | Chondroitin sulfate synthase 3 | CHSY3 | 0.82 |
| Q8WVM0 | Dimethyladenosine transferase 1, mitochondrial | TFB1M | 0.82 |
| Q07889 | Son of sevenless homolog 1 | SOS1 | 0.82 |
| Q6Y7W6 | GRB10-interacting GYF protein 2 | GIGYF2 | 0.82 |
| Q8TD16 | Protein bicaudal D homolog 2 | BICD2 | 0.82 |
| Q9BQ39 | ATP-dependent RNA helicase DDX50 | DDX50 | 0.82 |
| Q9H814 | Phosphorylated adapter RNA export protein | PHAX | 0.82 |
| Q8WVX3 | Uncharacterized protein C4orf3 | C4orf3 | 0.82 |
| Q8WU90 | Zinc finger CCCH domain-containing protein 15 | ZC3H15 | 0.82 |
| Q9ULW3 | Activator of basal transcription 1 | ABT1 | 0.82 |
| Q8WVR3 | Microtubule-associated protein 11 | MAP11 | 0.82 |
| P36873 | Serine/threonine-protein phosphatase PP1-gamma catalytic subunit | PPP1CC | 0.82 |
| O43294 | Transforming growth factor beta-1-induced transcript 1 protein | TGFB1I1 | 0.82 |
| Q12849 | G-rich sequence factor 1 | GRSF1 | 0.82 |
| Q9Y5J1 | U3 small nucleolar RNA-associated protein 18 homolog | UTP18 | 0.82 |
| P03956 | Interstitial collagenase | MMP1 | 0.82 |
| Q96EK5 | KIF-binding protein | KIFBP | 0.82 |
| P42684 | Tyrosine-protein kinase ABL2 | ABL2 | 0.82 |
| P52292 | Importin subunit alpha-1 | KPNA2 | 0.82 |
| P15924 | Desmoplakin | DSP | 0.81 |
| Q92979 | Ribosomal RNA small subunit methyltransferase NEP1 | EMG1 | 0.81 |
| Q9P0P0 | E3 ubiquitin-protein ligase RNF181 | RNF181 | 0.81 |
| Q9Y4F4 | TOG array regulator of axonemal microtubules protein 1 | TOGARAM1 | 0.81 |
| P01023 | Alpha-2-macroglobulin | A2M | 0.81 |
| Q7Z5Q1 | Cytoplasmic polyadenylation element-binding protein 2 | CPEB2 | 0.81 |
| Q8N1I8 | Putative uncharacterized protein encoded by CACTIN-AS1 | CACTIN-AS1 | 0.81 |
| Q06732 | Zinc finger protein 33B | ZNF33B | 0.81 |
| Q96EB1 | Elongator complex protein 4 | ELP4 | 0.81 |
| Q8NDD1 | Uncharacterized protein C1orf131 | C1orf131 | 0.81 |
| Q9NUQ3 | Gamma-taxilin | TXLNG | 0.81 |
| O00212 | Rho-related GTP-binding protein RhoD | RHOD | 0.81 |
| Q0VAA5 | PI-PLC X domain-containing protein 2 | PLCXD2 | 0.81 |
| O00148 | ATP-dependent RNA helicase DDX39A | DDX39A | 0.81 |
| Q96F63 | Coiled-coil domain-containing protein 97 | CCDC97 | 0.81 |
| Q9UBB4 | Ataxin-10 | ATXN10 | 0.81 |
| Q4LDG9 | Dynein light chain 1, axonemal | DNAL1 | 0.81 |
| Q8IZQ5 | Selenoprotein H | SELENOH | 0.81 |
| O00762 | Ubiquitin-conjugating enzyme E2 C | UBE2C | 0.81 |
| P47895 | Aldehyde dehydrogenase family 1 member A3 | ALDH1A3 | 0.81 |
| Q86WJ1 | Chromodomain-helicase-DNA-binding protein 1-like | CHD1L | 0.81 |
| Q9NRX1 | RNA-binding protein PNO1 | PNO1 | 0.81 |
| Q7L9L4 | MOB kinase activator 1B | MOB1B | 0.81 |
| Q96JI7 | Spatacsin | SPG11 | 0.81 |
| P28347 | Transcriptional enhancer factor TEF-1 | TEAD1 | 0.80 |
| Q7Z7F0 | KH homology domain-containing protein 4 | KHDC4 | 0.80 |
| P07711 | Cathepsin L1 | CTSL | 0.80 |
| P43235 | Cathepsin K | CTSK | 0.80 |
| Q96S16 | JmjC domain-containing protein 8 | JMJD8 | 0.80 |
| Q9UG63 | ATP-binding cassette sub-family F member 2 | ABCF2 | 0.80 |
| Q13895 | Bystin | BYSL | 0.80 |
| Q15653 | NF-kappa-B inhibitor beta | NFKBIB | 0.80 |
| Q7Z4H7 | HAUS augmin-like complex subunit 6 | HAUS6 | 0.80 |
| P25205 | DNA replication licensing factor MCM3 | MCM3 | 0.80 |
| A4D1E9 | GTP-binding protein 10 | GTPBP10 | 0.80 |
| P33991 | DNA replication licensing factor MCM4 | MCM4 | 0.80 |
| Q9HB40 | Retinoid-inducible serine carboxypeptidase | SCPEP1 | 0.80 |
| Q14012 | Calcium/calmodulin-dependent protein kinase type 1 | CAMK1 | 0.80 |
| Q9UDY4 | DnaJ homolog subfamily B member 4 | DNAJB4 | 0.80 |
| Q9BTE3 | Mini-chromosome maintenance complex-binding protein | MCMBP | 0.80 |
| P13533 | Myosin-6 | MYH6 | 0.80 |
| Q8IWC1 | MAP7 domain-containing protein 3 | MAP7D3 | 0.80 |
| P61024 | Cyclin-dependent kinases regulatory subunit 1 | CKS1B | 0.80 |
| Q9BV57 | 1,2-dihydroxy-3-keto-5-methylthiopentene dioxygenase | ADI1 | 0.79 |
| P31689 | DnaJ homolog subfamily A member 1 | DNAJA1 | 0.79 |
| Q9UJK0 | Ribosome biogenesis protein TSR3 homolog | TSR3 | 0.79 |
| Q8NDC4 | MORN repeat-containing protein 4 | MORN4 | 0.79 |
| Q96D09 | G-protein coupled receptor-associated sorting protein 2 | GPRASP2 | 0.79 |
| P06493 | Cyclin-dependent kinase 1 | CDK1 | 0.79 |
| Q71RC2 | La-related protein 4 | LARP4 | 0.79 |
| Q68DA7 | Formin-1 | FMN1 | 0.79 |
| Q9Y5Q0 | Fatty acid desaturase 3 | FADS3 | 0.79 |
| A0MZ66 | Shootin-1 | SHTN1 | 0.79 |
| Q14669 | E3 ubiquitin-protein ligase TRIP12 | TRIP12 | 0.79 |
| P41223 | Protein BUD31 homolog | BUD31 | 0.79 |
| P84243 | Histone H3.3 | H3-3A | 0.79 |
| A6ZKI3 | Retrotransposon Gag-like protein 8C | RTL8C | 0.79 |
| Q9Y3B9 | RRP15-like protein | RRP15 | 0.79 |
| P0CG29 | Glutathione S-transferase theta-2 | GSTT2 | 0.78 |
| Q9NXV2 | BTB/POZ domain-containing protein KCTD5 | KCTD5 | 0.78 |
| P13196 | 5-aminolevulinate synthase, nonspecific, mitochondrial | ALAS1 | 0.78 |
| Q99615 | DnaJ homolog subfamily C member 7 | DNAJC7 | 0.78 |
| Q9H0K6 | Pseudouridylate synthase 7 homolog-like protein | PUS7L | 0.78 |
| Q96F86 | Enhancer of mRNA-decapping protein 3 | EDC3 | 0.78 |
| Q9UPU9 | Protein Smaug homolog 1 | SAMD4A | 0.78 |
| Q14566 | DNA replication licensing factor MCM6 | MCM6 | 0.78 |
| Q16623 | Syntaxin-1A | STX1A | 0.78 |
| P10912 | Growth hormone receptor | GHR | 0.78 |
| Q9BY42 | Replication termination factor 2 | RTF2 | 0.78 |
| P39748 | Flap endonuclease 1 | FEN1 | 0.78 |
| Q8WVX9 | Fatty acyl-CoA reductase 1 | FAR1 | 0.78 |
| Q96EY4 | Translation machinery-associated protein 16 | TMA16 | 0.78 |
| Q5SW79 | Centrosomal protein of 170 kDa | CEP170 | 0.78 |
| Q9NRM1 | Enamelin | ENAM | 0.78 |
| P05546 | Heparin cofactor 2 | SERPIND1 | 0.78 |
| O14807 | Ras-related protein M-Ras | MRAS | 0.78 |
| Q7Z7A1 | Centriolin | CNTRL | 0.78 |
| Q96P53 | WD repeat and FYVE domain-containing protein 2 | WDFY2 | 0.78 |
| Q9UMY1 | Nucleolar protein 7 | NOL7 | 0.78 |
| Q9UEE5 | Serine/threonine-protein kinase 17A | STK17A | 0.78 |
| Q8N556 | Actin filament-associated protein 1 | AFAP1 | 0.77 |
| Q9C0D4 | Zinc finger protein 518B | ZNF518B | 0.77 |
| O00300 | Tumor necrosis factor receptor superfamily member 11B | TNFRSF11B | 0.77 |
| Q9BY76 | Angiopoietin-related protein 4 | ANGPTL4 | 0.77 |
| P55061 | Bax inhibitor 1 | TMBIM6 | 0.77 |
| Q9NYP7 | Elongation of very long chain fatty acids protein 5 | ELOVL5 | 0.77 |
| O95229 | ZW10 interactor | ZWINT | 0.77 |
| Q9UNZ5 | Leydig cell tumor 10 kDa protein homolog | C19orf53 | 0.77 |
| Q6AWC2 | Protein WWC2 | WWC2 | 0.77 |
| Q15785 | Mitochondrial import receptor subunit TOM34 | TOMM34 | 0.77 |
| O14929 | Histone acetyltransferase type B catalytic subunit | HAT1 | 0.77 |
| Q9ULX3 | RNA-binding protein NOB1 | NOB1 | 0.77 |
| Q9NWB7 | Intraflagellar transport protein 57 homolog | IFT57 | 0.77 |
| P17844 | Probable ATP-dependent RNA helicase DDX5 | DDX5 | 0.77 |
| Q8IZ73 | RNA pseudouridylate synthase domain-containing protein 2 | RPUSD2 | 0.76 |
| P01008 | Antithrombin-III | SERPINC1 | 0.76 |
| Q8IV48 | 3'-5' exoribonuclease 1 | ERI1 | 0.76 |
| P29279 | CCN family member 2 | CCN2 | 0.76 |
| Q8NB46 | Serine/threonine-protein phosphatase 6 regulatory ankyrin repeat subunit C | ANKRD52 | 0.76 |
| Q9H3C7 | Gametogenetin-binding protein 2 | GGNBP2 | 0.76 |
| P46013 | Proliferation marker protein Ki-67 | MKI67 | 0.76 |
| O94886 | CSC1-like protein 1 | TMEM63A | 0.76 |
| Q12851 | Mitogen-activated protein kinase kinase kinase kinase 2 | MAP4K2 | 0.76 |
| Q9BVS4 | Serine/threonine-protein kinase RIO2 | RIOK2 | 0.76 |
| Q8N9A8 | Nuclear envelope phosphatase-regulatory subunit 1 | CNEP1R1 | 0.76 |
| P57076 | Cilia- and flagella-associated protein 298 | CFAP298 | 0.76 |
| O43709 | Probable 18S rRNA (guanine-N(7))-methyltransferase | BUD23 | 0.75 |
| Q96BX8 | MOB kinase activator 3A | MOB3A | 0.75 |
| Q8N5G2 | Macoilin | MACO1 | 0.75 |
| Q9NZ63 | Telomere length and silencing protein 1 homolog | C9orf78 | 0.75 |
| Q9H0Y0 | Ubiquitin-like-conjugating enzyme ATG10 | ATG10 | 0.75 |
| Q9NP84 | Tumor necrosis factor receptor superfamily member 12A | TNFRSF12A | 0.75 |
| Q8IVL0 | Neuron navigator 3 | NAV3 | 0.75 |
| Q96KB5 | Lymphokine-activated killer T-cell-originated protein kinase | PBK | 0.75 |
| Q6IQ20 | N-acyl-phosphatidylethanolamine-hydrolyzing phospholipase D | NAPEPLD | 0.75 |
| Q96CS2 | HAUS augmin-like complex subunit 1 | HAUS1 | 0.74 |
| Q86XK3 | Swi5-dependent recombination DNA repair protein 1 homolog | SFR1 | 0.74 |
| O75916 | Regulator of G-protein signaling 9 | RGS9 | 0.74 |
| P15408 | Fos-related antigen 2 | FOSL2 | 0.74 |
| Q96T88 | E3 ubiquitin-protein ligase UHRF1 | UHRF1 | 0.74 |
| O75190 | DnaJ homolog subfamily B member 6 | DNAJB6 | 0.74 |
| Q9Y6V7 | Probable ATP-dependent RNA helicase DDX49 | DDX49 | 0.73 |
| P26358 | DNA (cytosine-5)-methyltransferase 1 | DNMT1 | 0.73 |
| Q96GM8 | Target of EGR1 protein 1 | TOE1 | 0.73 |
| Q6P5R6 | 60S ribosomal protein L22-like 1 | RPL22L1 | 0.73 |
| Q9Y385 | Ubiquitin-conjugating enzyme E2 J1 | UBE2J1 | 0.73 |
| Q8N543 | Prolyl 3-hydroxylase OGFOD1 | OGFOD1 | 0.73 |
| Q16850 | Lanosterol 14-alpha demethylase | CYP51A1 | 0.73 |
| Q9UNQ2 | Probable dimethyladenosine transferase | DIMT1 | 0.72 |
| Q9NR30 | Nucleolar RNA helicase 2 | DDX21 | 0.72 |
| P07992 | DNA excision repair protein ERCC-1 | ERCC1 | 0.72 |
| P00747 | Plasminogen | PLG | 0.72 |
| Q8IZJ3 | C3 and PZP-like alpha-2-macroglobulin domain-containing protein 8 | CPAMD8 | 0.72 |
| P07951 | Tropomyosin beta chain | TPM2 | 0.72 |
| Q9BT25 | HAUS augmin-like complex subunit 8 | HAUS8 | 0.72 |
| Q9H0S4 | Probable ATP-dependent RNA helicase DDX47 | DDX47 | 0.72 |
| O60870 | DNA/RNA-binding protein KIN17 | KIN | 0.72 |
| P80188 | Neutrophil gelatinase-associated lipocalin | LCN2 | 0.72 |
| Q9H8M2 | Bromodomain-containing protein 9 | BRD9 | 0.71 |
| Q92896 | Golgi apparatus protein 1 | GLG1 | 0.71 |
| Q15413 | Ryanodine receptor 3 | RYR3 | 0.71 |
| Q05D32 | CTD small phosphatase-like protein 2 | CTDSPL2 | 0.71 |
| Q9H2J4 | Phosducin-like protein 3 | PDCL3 | 0.71 |
| Q9Y248 | DNA replication complex GINS protein PSF2 | GINS2 | 0.71 |
| Q9UQR1 | Zinc finger protein 148 | ZNF148 | 0.71 |
| Q9NQ30 | Endothelial cell-specific molecule 1 | ESM1 | 0.71 |
| Q5JUR7 | Testis-expressed protein 30 | TEX30 | 0.71 |
| P0C1Z6 | TCF3 fusion partner | TFPT | 0.71 |
| Q8TCB0 | Interferon-induced protein 44 | IFI44 | 0.70 |
| P24941 | Cyclin-dependent kinase 2 | CDK2 | 0.70 |
| A6NED2 | RCC1 domain-containing protein 1 | RCCD1 | 0.70 |
| Q9UIJ5 | Palmitoyltransferase ZDHHC2 | ZDHHC2 | 0.70 |
| P24385 | G1/S-specific cyclin-D1 | CCND1 | 0.69 |
| P04198 | N-myc proto-oncogene protein | MYCN | 0.69 |
| Q15054 | DNA polymerase delta subunit 3 | POLD3 | 0.69 |
| P41218 | Myeloid cell nuclear differentiation antigen | MNDA | 0.69 |
| O95497 | Pantetheinase | VNN1 | 0.69 |
| P11388 | DNA topoisomerase 2-alpha | TOP2A | 0.68 |
| Q96I51 | RCC1-like G exchanging factor-like protein | RCC1L | 0.68 |
| Q8NEM2 | SHC SH2 domain-binding protein 1 | SHCBP1 | 0.68 |
| Q4U2R6 | 39S ribosomal protein L51, mitochondrial | MRPL51 | 0.67 |
| Q15493 | Regucalcin | RGN | 0.67 |
| Q00537 | Cyclin-dependent kinase 17 | CDK17 | 0.67 |
| P02458 | Collagen alpha-1(II) chain | COL2A1 | 0.67 |
| Q9HBU6 | Ethanolamine kinase 1 | ETNK1 | 0.67 |
| Q13751 | Laminin subunit beta-3 | LAMB3 | 0.67 |
| Q9HCK1 | DBF4-type zinc finger-containing protein 2 | ZDBF2 | 0.66 |
| P09493 | Tropomyosin alpha-1 chain | TPM1 | 0.66 |
| P47974 | mRNA decay activator protein ZFP36L2 | ZFP36L2 | 0.65 |
| Q9BQP7 | Mitochondrial genome maintenance exonuclease 1 | MGME1 | 0.65 |
| A3KN83 | Protein strawberry notch homolog 1 | SBNO1 | 0.65 |
| O75326 | Semaphorin-7A | SEMA7A | 0.64 |
| P19823 | Inter-alpha-trypsin inhibitor heavy chain H2 | ITIH2 | 0.64 |
| Q9HBM1 | Kinetochore protein Spc25 | SPC25 | 0.63 |
| Q92908 | Transcription factor GATA-6 | GATA6 | 0.63 |
| P53814 | Smoothelin | SMTN | 0.63 |
| P06400 | Retinoblastoma-associated protein | RB1 | 0.62 |
| Q96GS6 | Alpha/beta hydrolase domain-containing protein 17A | ABHD17A | 0.62 |
| Q53GA4 | Pleckstrin homology-like domain family A member 2 | PHLDA2 | 0.62 |
| P31350 | Ribonucleoside-diphosphate reductase subunit M2 | RRM2 | 0.62 |
| Q96RS0 | Trimethylguanosine synthase | TGS1 | 0.61 |
| P15407 | Fos-related antigen 1 | FOSL1 | 0.61 |
| Q13163 | Dual specificity mitogen-activated protein kinase kinase 5 | MAP2K5 | 0.60 |
| Q99856 | AT-rich interactive domain-containing protein 3A | ARID3A | 0.60 |
| Q96GV9 | UNC119-binding protein C5orf30 | C5orf30 | 0.60 |
| Q8N441 | Fibroblast growth factor receptor-like 1 | FGFRL1 | 0.59 |
| Q9ULW0 | Targeting protein for Xklp2 | TPX2 | 0.59 |
| Q8N5A5 | Zinc finger CCCH-type with G patch domain-containing protein | ZGPAT | 0.58 |
| O60427 | Acyl-CoA (8-3)-desaturase | FADS1 | 0.58 |
| Q12772 | Sterol regulatory element-binding protein 2 | SREBF2 | 0.58 |
| P04818 | Thymidylate synthase | TYMS | 0.58 |
| Q14679 | Tubulin polyglutamylase TTLL4 | TTLL4 | 0.57 |
| Q8NI35 | InaD-like protein | PATJ | 0.57 |
| P04183 | Thymidine kinase, cytosolic | TK1 | 0.55 |
| P02549 | Spectrin alpha chain, erythrocytic 1 | SPTA1 | 0.53 |
| O43164 | E3 ubiquitin-protein ligase Praja-2 | PJA2 | 0.53 |
| Q14624 | Inter-alpha-trypsin inhibitor heavy chain H4 | ITIH4 | 0.51 |
| P0C0L4 | Complement C4-A | C4A | 0.51 |
| Q99538 | Legumain | LGMN | 0.49 |
| Q06033 | Inter-alpha-trypsin inhibitor heavy chain H3 | ITIH3 | 0.49 |
| Q494V2 | Cilia- and flagella-associated protein 100 | CFAP100 | 0.49 |
| Q99541 | Perilipin-2 | PLIN2 | 0.49 |
| P02771 | Alpha-fetoprotein | AFP | 0.48 |
| P02768 | Serum albumin | ALB | 0.48 |
| Q53T94 | TATA box-binding protein-associated factor RNA polymerase I subunit B | TAF1B | 0.48 |
| Q04756 | Hepatocyte growth factor activator | HGFAC | 0.47 |
| P02787 | Serotransferrin | TF | 0.39 |
| P02790 | Hemopexin | HPX | 0.31 |


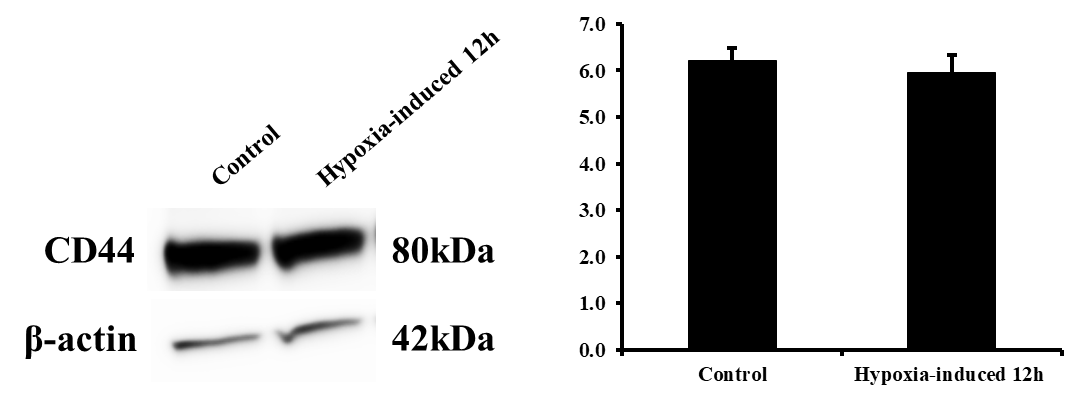


Figure S1 CD44 protein expressions were analyzed by western blotting and quantified by densitometry. CD44 was no significant difference between the hypoxia-induced 12 hours group and the control group in hBMSCs. n=3.


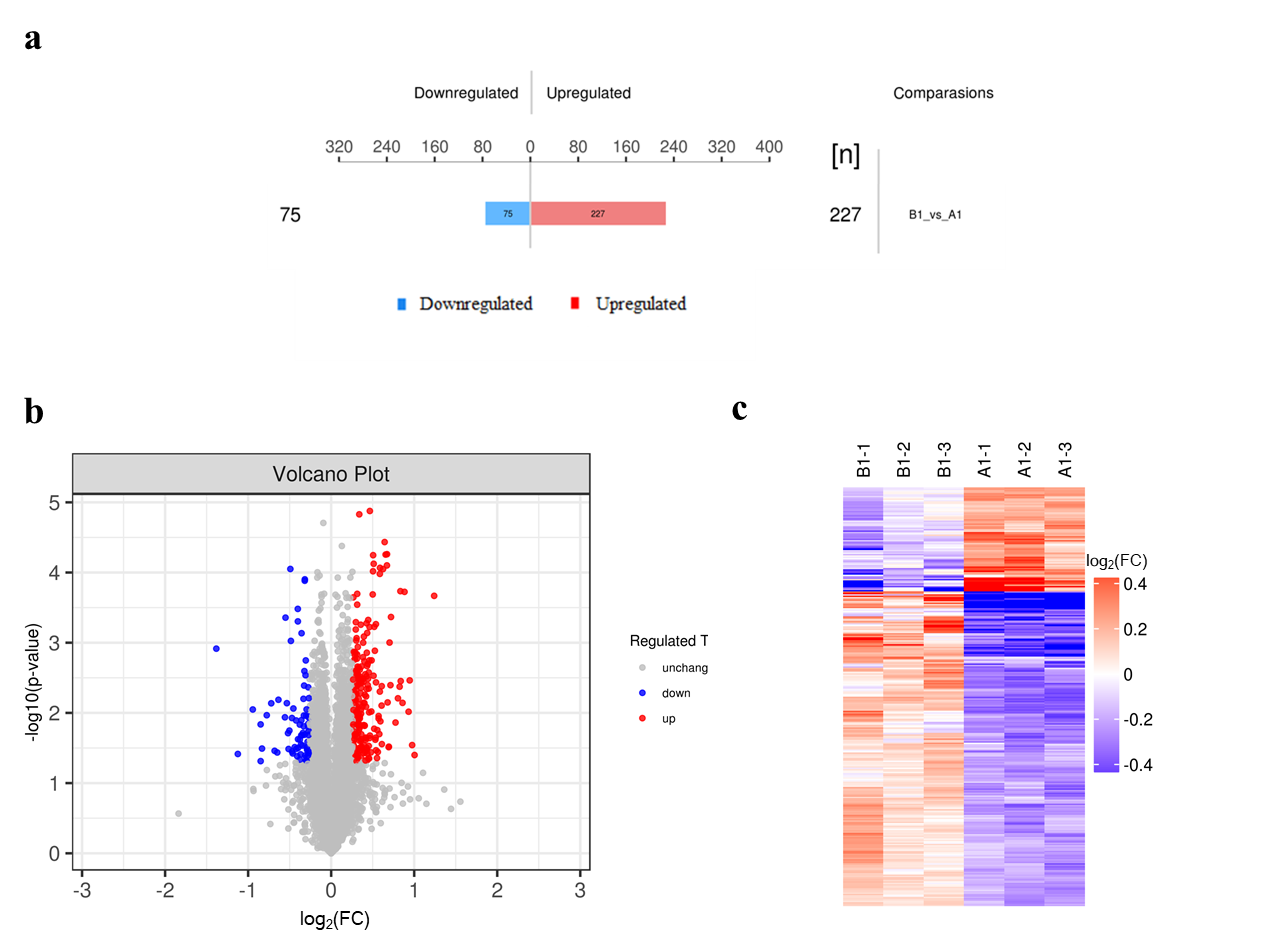


Figure S2 Profiling of differentially expressed proteins between the hypoxia-treatment 4 hours group and the control group. a. The up- (red) or down-regulated (blue) proteins were displayed in hypoxia-treatment 4 hours groups. 302 significantly differentially expressed proteins were identified, of which 227 were up-regulated and 75 were down-regulated after hypoxia induction for 4 hours (group B1), compared to the control group (group A1). b. Volcano plot showing the up- (red) or down-regulated (purple) proteins between the hypoxia-induced 4 hour group (group B1) and the control group (group A1). c. The hierarchical clustering heat map of differentially expressed proteins between the hypoxia-induced 4 hour group (group B1) and the control group (group A1). Each column represents a set of samples, while each row represents a protein in the figure. The red represents significant increase protein, while purple represents significant lower protein in the heat map, gray part means quantitative information without protein.


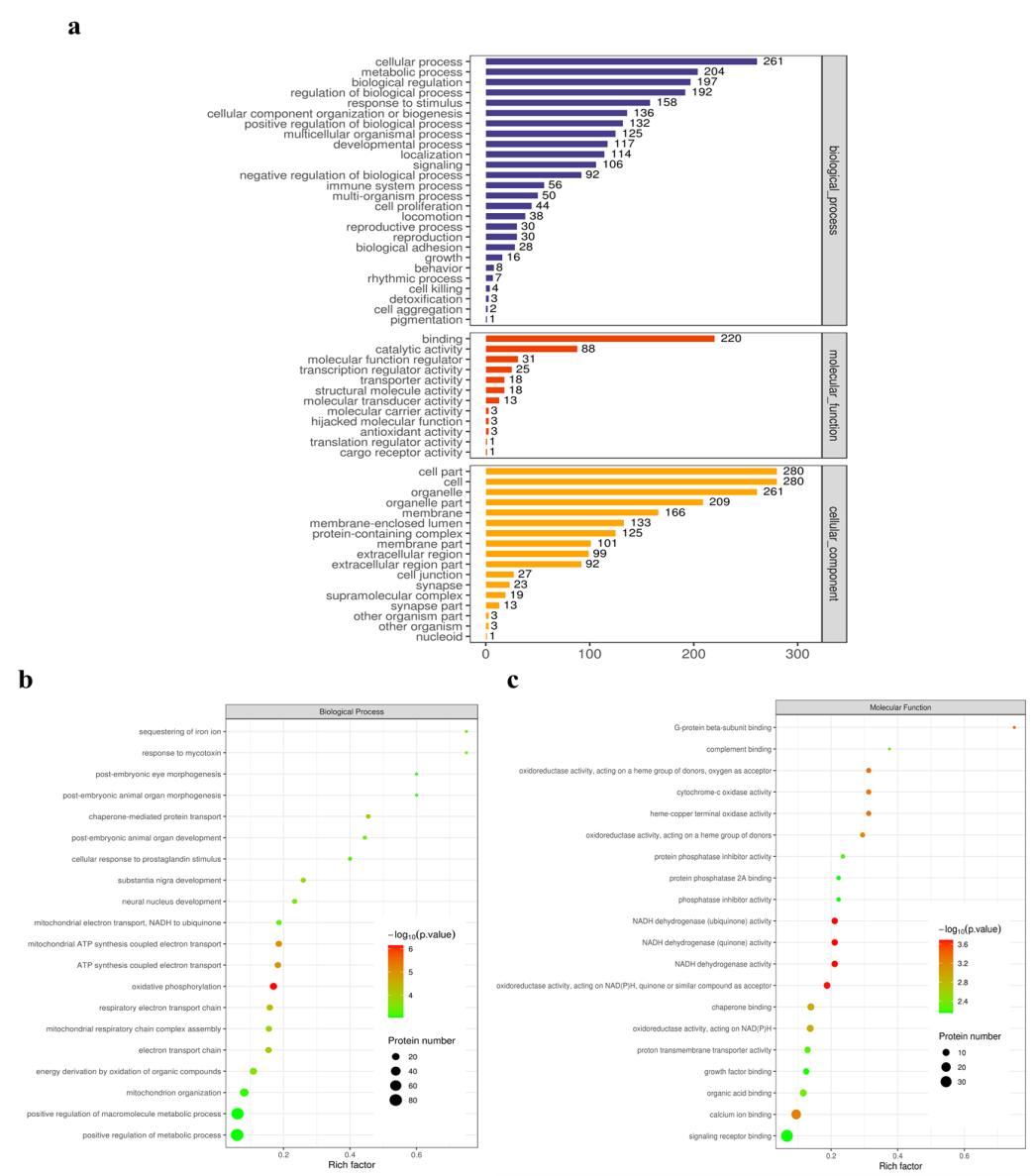


Figure S3. Profiling of differentially expressed proteins using GO analysis between the hypoxia-treatment 4 hours group and the control group. a. GO annotated statistics of differentially expressed proteins. The Gene Ontology categories of differentially expressed proteins based on biological process(BP),molecular function(MF) and cellular component (CC) between the hypoxia-induced 4 hour group and the control group. b. As for BP, the up-regulated (Red) differentially expressed proteins were involved in oxidative phosphorylation, ATP synthesis coupled electron transport, mitochondrial ATP synthesis coupled electron transport. The down-regulated (green) proteins were enriched in positive regulation of metabolic process, positive regulation of macromolecule metabolic process, mitochondrion organization, energy derivation by oxidation of organic compounds, mitochondrial electron transport (NADH to ubiquinone), neural nucleus development. c. In terms of MF, the up-regulated (red) proteins were mainly annotated in oxidoreductase activity, acting on NAD(P)H, quinone or similar compound as acceptor, NADH dehydrogenase activity, NADH dehydrogenase(quinone) activity, NADH dehydrogenase(ubiquinone) activity. Down-regulated (green) proteins were enriched in signaling receptor binding, proton transmembrane transporter activity, growth factor binding, organic acid binding, phosphatase inhibitor activity, protein phosphatase 2A binding, protein phosphatase inhibitor activity.


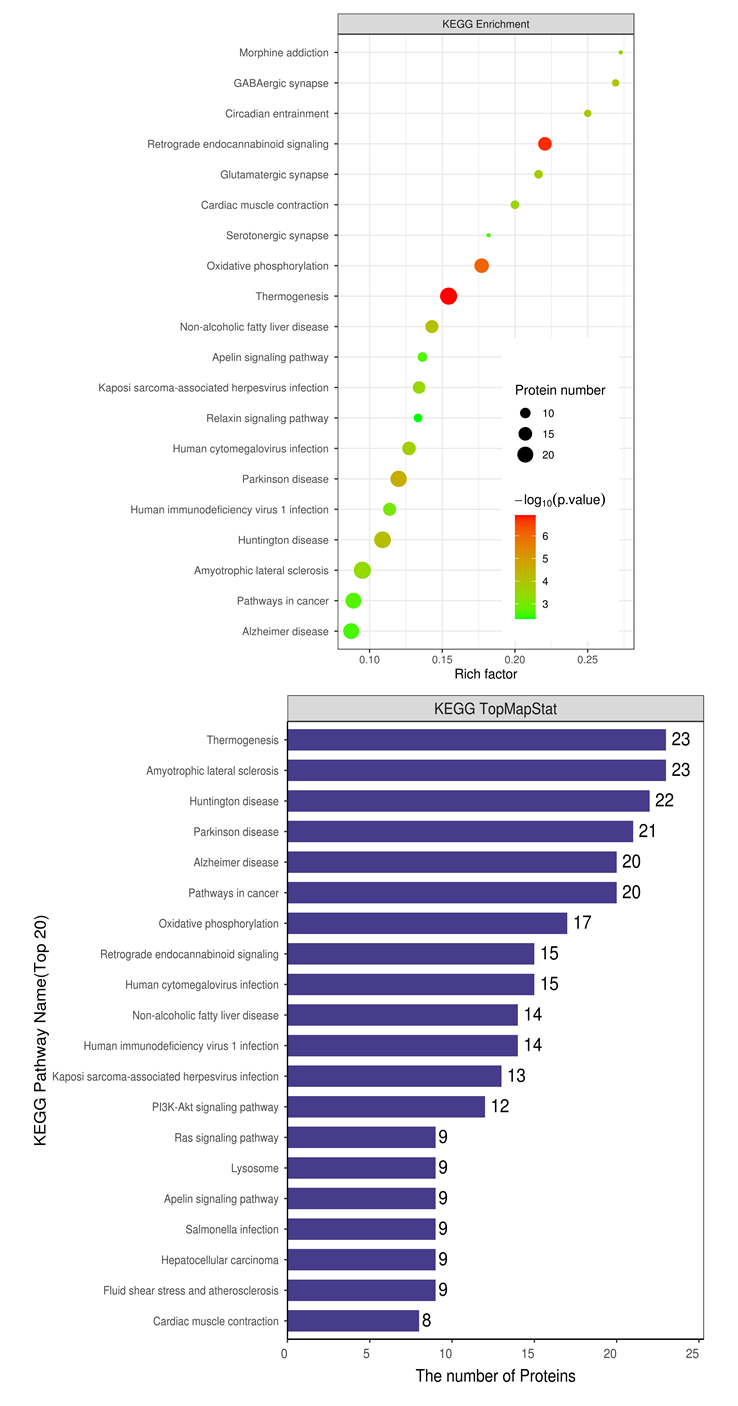


Figure S4 KEGG pathway analysis of hBMSCs between the hypoxia-induced 4 hours group and the control group. a. KEGG pathway enrichment analysis of differentially expressed proteins from cells treated with or without hypoxia. The up-regulated proteins were mainly involved in Thermogenesis, Retrograde endocannabinoid signaling, Oxidative phosphorylation, whereas the down-regulated were mainly involved in Alzheimer disease, pathways in cancer, Relaxin signaling pathway, Apelin signaling pathway. b. KEGG pathway annotation statistics of differentially expressed proteins (Top20). The signaling pathway annotation of differentially expressed proteins were critically related with Thermogenesis, Amyotrophic lateral sclerosis, Huntington disease, Parkinson disease, Alzheimer disease, pathways in cancer, Oxidative phosphorylation.
